# An integrative genome-wide transcriptome reveals that candesartan is neuroprotective and a candidate therapeutic for Alzheimer’s disease

**DOI:** 10.1186/s13195-015-0167-5

**Published:** 2016-01-28

**Authors:** Abdel G. Elkahloun, Roman Hafko, Juan M. Saavedra

**Affiliations:** Comparative genomics and Cancer Genetics Branch, National Human Genome Research Institute, National Institutes of Health, Bethesda, MD 20892 USA; Section on Pharmacology, National Institute of Mental Health, National Institutes of Health, Bethesda, MD 20892 USA; Department of Pharmacology and Physiology, Georgetown University Medical Center, SE402 Med/Dent, 3900 Reservoir Road, Washington, DC 20057 USA

**Keywords:** Alzheimer’s disease, Neurodegenerative disorders, Glutamate excitotoxicity, Neuroprotection, Angiotensin II receptor blockers, IPA analysis, GEO database, GSEA

## Abstract

**Background:**

Alzheimer’s disease is the most frequent age-related dementia, and is currently without treatment. To identify possible targets for early therapeutic intervention we focused on glutamate excitotoxicity, a major early pathogenic factor, and the effects of candesartan, an angiotensin receptor blocker of neuroprotective efficacy in cell cultures and rodent models of Alzheimer’s disease. The overall goal of the study was to determine whether gene analysis of drug effects in a primary neuronal culture correlate with alterations in gene expression in Alzheimer’s disease, thus providing further preclinical evidence of beneficial therapeutic effects.

**Methods:**

Primary neuronal cultures were treated with candesartan at neuroprotective concentrations followed by excitotoxic glutamate amounts. We performed genome-wide expression profile analysis and data evaluation by ingenuity pathway analysis and gene set enrichment analysis, compared with alterations in gene expression from two independent published datasets identified by microarray analysis of postmortem hippocampus from Alzheimer’s disease patients. Preferential expression in cerebrovascular endothelial cells or neurons was analyzed by comparison to published gene expression in these cells isolated from human cortex by laser capture microdissection.

**Results:**

Candesartan prevented glutamate upregulation or downregulation of several hundred genes in our cultures. Ingenuity pathway analysis and gene set enrichment analysis revealed that inflammation, cardiovascular disease and diabetes signal transduction pathways and amyloid β metabolism were major components of the neuronal response to glutamate excitotoxicity. Further analysis showed associations of glutamate-induced changes in the expression of several hundred genes, normalized by candesartan, with similar alterations observed in hippocampus from Alzheimer’s disease patients. Gene analysis of neurons and cerebrovascular endothelial cells obtained by laser capture microdissection revealed that genes up- and downregulated by glutamate were preferentially expressed in endothelial cells and neurons, respectively.

**Conclusions:**

Our data may be interpreted as evidence of direct candesartan neuroprotection beyond its effects on blood pressure, revealing common and novel disease mechanisms that may underlie the in vitro gene alterations reported here and glutamate-induced cell injury in Alzheimer’s disease. Our observations provide novel evidence for candesartan neuroprotection through early molecular mechanisms of injury in Alzheimer’s disease, supporting testing this compound in controlled clinical studies in the early stages of the illness.

**Electronic supplementary material:**

The online version of this article (doi:10.1186/s13195-015-0167-5) contains supplementary material, which is available to authorized users.

## Background

Alzheimer’s disease is the most common age-related dementia and a major and increasing burden for our society [1]. This disorder is currently without treatment, and therapies to ameliorate alterations in amyloid beta (Aβ) or tau metabolism, initiated too late in the disease course, have proven disappointing [2]. For this reason the search for early pathogenic mechanisms susceptible to therapeutic intervention is a medical necessity.

Emerging evidence indicates a role for multifactorial mechanisms involved in the early stages of the disease and preceding diagnosis. These mechanisms include genetic factors, possibly of cumulative impact [3], risk factors, such as cardiovascular disease and diabetes [4, 5], alterations in the microvasculature [4–6], chronic dysregulated inflammation [6, 7] and glutamate-mediated excitotoxicity [3, 8–10].

In the present report we focused on glutamate excitotoxicity. Glutamate is the predominant excitatory neurotransmitter in the mammalian brain and participates in mechanisms associated with long-term potentiation and synaptic plasticity [11]. Excessive production and release of glutamate, however, leads to neuronal injury and is a major pathogenic factor in many acute and chronic brain conditions, including Alzheimer’s disease [3, 8–10, 12].

In our search for novel compounds with neuroprotective effects against glutamate neurotoxicity, we focused in a class of compounds, the angiotensin receptor blockers (ARBs) or sartans, that effectively blocks the physiological AT1 receptor (AT1R) and therefore the effects of angiotensin II, the main active factor of the renin–angiotensin system [13] both in the periphery and the brain [14]. Excessive peripheral AT1R activity associates with hypertension, heart and kidney failure, peripheral vascular and tissue inflammation, and metabolic abnormalities such as insulin resistance [15–17]. Consequently, the use of sartans, because of their beneficial effects on inflammatory and metabolic alterations beyond their effect on blood pressure control, has become a cornerstone for the treatment of cardiovascular and chronic kidney disease [18]. In turn, increased brain AT1R stimulation associates with brain ischemia, blood–brain barrier breakdown, Aβ production and toxicity, brain inflammation, traumatic brain injury and glutamate excitotoxicity, risk factors leading to neuronal injury, cognitive decline, and the incidence and progression of neurodegenerative diseases [19–26]. For this reason it is not surprising that sartans have been found to be effective neuroprotective compounds. In vitro experiments demonstrated that sartans ameliorate neuronal injury produced by glutamate excitotoxicity and high levels of interleukin (IL)-1β, and microglia activation as a result of systemic administration of bacterial endotoxin (lipopolysaccharide (LPS)) [25, 27–29]. In rodent models of Alzheimer’s disease, sartans (candesartan, losartan, valsartan and telmisartan) ameliorate all risk factors for human Alzheimer’s disease, including protecting cerebral blood flow and cognition during stroke, decreasing inflammation and Aβ neurotoxicity, and reducing traumatic brain injury [24, 26, 27, 30–37]. Furthermore, clinical studies indicate that ARBs protect cognition after stroke and during aging [15, 22, 38, 39], and cohort analyses reveal that treatment of hypertension with sartans significantly reduces the incidence and progression of Alzheimer’s disease [40, 41].

To clarify the role of glutamate excitotoxicity, we used primary rat cerebellar granule cells (CGCs) in vitro. This is a well-characterized and reliable primary neuronal model to analyze mechanisms and excitotoxic neuronal damage and neuroprotection [42, 43]. Although in humans CGCs are not primary targets for Alzheimer’s disease, rat CGCs are very sensitive to glutamate excitotoxicity, a major early injury factor in this illness, and are extensively used in Alzheimer’s disease research [44–46]. We selected the ARB candesartan for our study because of its demonstrated neuroprotective effects on cultured primary cortical neurons, microglia and cerebrovascular endothelial cells, and its amelioration of brain inflammation in vivo [27] including reducing glutamate-induced apoptosis in cultured CGCs [25].

Our study was initially designed to provide mechanistic insight into the potential targets and pathways that may underlie glutamate-induced cell injury and its possible reversal by the neuroprotective action of candesartan. To this aim, we performed genome-wide expression analysis and evaluated the data with several pathway analysis programs: ingenuity pathway analysis (IPA), gene set enrichment analysis (GSEA) [47, 48] and Kyoto encyclopedia of genes and genomes (KEGG).

The strong correlation of our findings with many signal transduction mechanisms and pathways associated with Alzheimer’s disease prompted us to determine whether there was an association between the changes in gene expression in our study with those found in postmortem brain samples from patients who suffered from Alzheimer’s disease. To this end, we compared our results with alterations in gene expression published in two independent microarray studies of hippocampal samples obtained postmortem from brains of patients diagnosed with Alzheimer’s disease. Because of evidence of cerebrovascular endothelial dysfunction in Alzheimer’s disease, we wanted to establish which of the genes altered in Alzheimer’s disease patients were predominantly expressed in cerebrovascular endothelial cells or in neurons. To clarify this point, we compared gene expressions altered in published Alzheimer’s disease patients with published analysis of predominant gene expression in human cerebrovascular endothelial cells and neurons obtained by laser capture microdissection from postmortem dorsolateral prefrontal cortex samples and then we looked at the effect of candesartan on these gene signatures in our CGC study.

## Methods

### Culture of primary neurons

Animal housing, handling and experimentation were in compliance with guidelines and protocol approval by the NIMH (NIH) Institutional Animal Care and Use Committee (protocol MH002762-17), and followed guidelines of the US National Institute of Health Guide for the Care and Use of Laboratory Animals published by the US National Academy of Sciences (http://oacu.od.nih.gov/regs/index.htm).

We used primary cultures of rat primary CGCs, which are very sensitive to glutamate excitotoxicity and extensively used in Alzheimer’s disease research [44–46]. CGCs were isolated from 8-day old Sprague Dawley rat pups (Charles Rivers Laboratories, Wilmington, MA, USA) as described previously [49, 50] which were euthanized by decapitation. Brains were dissected immediately and the cerebella were collected and placed in ice-cold Hank’s balanced salt solution (Invitrogen, Carlsbad, CA, USA). After removal of the meninges, the cerebella were dispersed into the same buffer containing 0.025 % trypsin (Invitrogen) and digested for 15 min at 37 °C. Trypsin digestion was stopped by adding the same volume of Dulbecco’s modified Eagle’s medium (Invitrogen), supplemented with 10 % fetal bovine serum (Invitrogen) and 0.1 mg/ml DNase I (Sigma-Aldrich, St. Louis, MO, USA). After gentle trituration, digested tissues were centrifuged at 1000 rpm for 5 min. The cell pellets were suspended in the complete Neurobasal culture medium supplemented with 2 % B27 (Invitrogen) and 0.5 mM GlutaMax (Invitrogen). After filtration through a 70 mm cell restrainer (BD Falcon, Vernon Hills, IL, USA), cells were plated at a density of 1 × 10^6^ cells/ml onto poly-L-lysine coated plates (Becton Dickinson and Company, Franklin Lakes, NJ, USA) or chamber glass slides (Nalge Nunc International, Naperville, IL, USA). Cultures were incubated in a humidified atmosphere of 5 % CO_2_ 95 % air at 37 °C. Cytosine arabinofuranoside (Invitrogen) (10 µl) was added to the cultures 24 h after plating to arrest the growth of non-neuronal cells. Cultures of 6 to 7 days in vitro were used in this study. Immunocytochemical validation with antimicrotubule-associated protein-2 antibody (EMD Millipore, Billerica, MA, USA) and 4-6-diamino-2-phenylindole (Invitrogen) revealed that more than 95 % of the cells in our culture system were neurons at the time of experiment (data not shown).

### Cell culture treatments

**Fig. 1:**
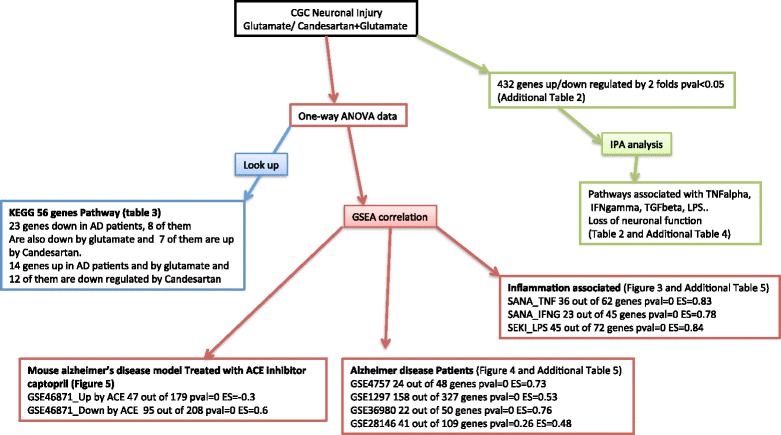
Flow chart for data analysis. *ACE* angiotensin converting enzyme, AD Alzheimer’s disease, *ANOVA* analysis of variance, *CGC* cerebellar granule cell, *ES* enrichment score, *GSEA* gene set enrichment analysis, *IFN* interferon, *IPA* ingenuity pathway analysis, *KEGG* Kyoto encyclopedia of genes and genomes, *LPS* lipopolysaccharide, *pval* p value, TGF transforming growth factor, *TNF* tumor necrosis factor

We performed microarray analysis of gene expression in four sets of rat primary neuronal cultures: controls; treated with candesartan at concentrations in the range of blood levels obtained in humans after oral administration [51]; exposed to excitotoxic glutamate concentrations [25]; and treated with candesartan before the exposure to glutamate. Excitotoxicity was induced by exposing cultures to 100 μM glutamate (Sigma-Aldrich) and pretreated for 1 h with vehicle (0.1 % saline and 0.1 N Na_2_CO_3_ at pH 7.4), or the AT1R blocker candesartan (10 μM) (Sigma-Aldrich) dissolved in 0.1 N Na_2_CO_3_, pH 7.4. After addition of candesartan, the compound was not removed and was present throughout the incubation. Candesartan and glutamate concentrations and timing of the experiments were selected on the basis of prior studies demonstrating protection of cultured neurons from inflammation and glutamate-induced injury [25, 27]. Figure 1 is a flow chart for data analysis.

### Gene expression analysis

Total RNA was extracted from CGC treated with vehicle, CGC treated with candesartan, CGC treated with glutamate and CGC treated with candesartan and glutamate. Each group consisted of five independent experiments. Standard procedures for labeling, hybridization, washing and staining were as per manufacturer’s recommendation (Affymetrix, Santa Clara, CA, USA). Briefly, the RNA was purified using a RiboPure Kit (Ambion, Austin, TX, USA) according to the manufacturer’s protocol. The quality and quantity of RNA were ensured using the Bioanalyzer (Agilent, Santa Clara, CA, USA) and NanoDrop (Thermo Scientific, Waltham, MA, USA), respectively. For RNA labeling, total RNA (300 ng) was used in conjunction with the Affymetrix-recommended protocol with the WT Plus Reagent Kit catalog #902280. The hybridization cocktail containing the fragmented and labeled complementary DNAs (cDNAs) was hybridized to the Rat GeneChip 2.0 ST chips. The chips were washed and stained by the Affymetrix Fluidics Station using the standard format and protocols from Affymetrix. The probe arrays were stained with streptavidin phycoerythrin solution (Molecular Probes, Carlsbad, CA, USA) and enhanced by using an antibody solution containing 0.5 mg/ml biotinylated anti-streptavidin (Vector Laboratories, Burlingame, CA, USA). The probe arrays were scanned using an Affymetrix Gene Chip Scanner 3000. Gene expression intensities were calculated using the Gene Chip Operating software 1.2 (Affymetrix). A GC-corrected robust multichip analysis (RMA) normalization model was used to correct for background, and nonspecific binding. All analyses were performed using Partek Genomics Suite (Fig. 1). The raw data is submitted to Gene Expression Omnibus (GEO) under accession GSE67036.

### Quantitative real-time polymerase chain reaction

Aliquots from samples from the same experiment were used for quantitative real-time polymerase chain reaction (qPCR) and for microarray analysis. For qPCR we studied three individual independent samples, and each sample was analyzed in triplicate.

To determine gene expression, total RNA was isolated as indicated using 1 ml TRIzol (Invitrogen), followed by purification using an RNeasy Mini kit (Qiagen, Valencia, CA, USA) according to the manufacturer’s instructions. Synthesis of cDNA was performed using 0.6 mg total RNA and Super-Script III first-Strand Synthesis kit (Invitrogen). qPCR was performed on DNA Engine Opticon (MJ Research, Waltham, MA, USA) with SYBR Green PCR Master Mix (Applied Biosystems, Foster City, CA, USA). qPCR was performed in a 20 μl reaction mixture containing 10 μl SYBR Green PCR Master Mix, 4 μl cDNA and 0.3 μM of each primer for a specific target. Primers for qPCR were synthesized by BioServe (Beltsville, MD, USA). The specific primers are listed in Additional file 1 (Table S1). The remaining reagents for RNA isolation and reverse transcription were from Invitrogen. The amplification conditions consisted of one denaturation/activation cycle at 95 °C for 10 min, followed by 45 cycles at 95 °C for 15 s and 60 °C for 60 s. Serial dilutions of cDNA from the same source as samples were used to obtain a standard curve. The individual targets for each sample were quantified by determining the cycle threshold and by comparison with the standard curve. The relative amount of the target mRNA was normalized with the housekeeping gene glyceraldehyde-3-phosphate dehydrogenase.

Multiple group comparisons for data obtained by qPCR were performed by one-way analysis of variance (ANOVA) followed by Newman-Keuls post-test. Statistical significance was determined using GraphPad Prism 5 Software (GraphPad Software, San Diego, CA, USA). In all cases, data are accepted as statistically significant given a probability value of ≤0.05.

### Datasets description and microarray data mining

To compare our data to published datasets, we used GSEA [52] (Fig. 1). The GSEA algorithm computes a ranked list of all genes from a microarray comparison between two conditions and identifies whether individual members of an a priori functionally defined gene set (black vertical bars) are enriched at either the top (red area) or bottom of the ranked genes (blue area) or randomly distributed across the whole ranked gene list, using a modified Kolmogorov-Smirnov statistic. These predefined gene sets are part of a functionally well-established and/or published pathway from databases such as KEGG [53], BioCarta [54], Reactome [55] and gene ontology. An enrichment score (green graph) is calculated based on the level to which a gene set is overrepresented at the top (positive correlation) or bottom (negative correlation) of the ranked gene list and is calculated as the maximum deviation from zero. Genes occurring at the very extreme (dark red or dark blue area) on either side of the ranked list are weighted more heavily compared with genes occurring in the middle (light red or light blue area) of the ranked gene list that contain genes that are not differentially expressed. Statistical significance is defined by the *p*-value, which is also adjusted for multiple hypothesis testing. A gene set-based permutation test of 1000 permutations was applied and genes were ranked according to Student’s *t* statistic. All other parameters were set to GSEA defaults [47, 48]. For a more comprehensive description of the GSEA [47, 48, 52] and the Broad Molecular Signatures Database v5.0 (MSigDB) [56] see [47, 48, 52]. The MSigDB actually consists of over 4000 different gene sets. Alternatively, we used microarray datasets from the GEO database [57] to derive gene sets that we then used for GSEA analysis. IPA [58] (Ingenuity Systems, Redwood City, CA, USA) was used to identify canonical pathways associated with the differentially expressed genes. All of the differentially expressed genes were included in the analysis.

Datasets from normal/Alzheimer’s disease whole hippocampus tissue comparisons (GSE1297 [59] GSE48350 [60], and GSE36980 [61]) and from laser capture microdissected normal neuronal/endothelial cell comparisons (GSE12679 [62] and GSE12293 [63]) were downloaded from NCBI’s GEO database [57] and imported into Partek Genomics Suite software (Partek, Inc., St. Louis, MI, USA) (Fig. 1). After RMA normalization, differential gene expression was accessed by one-way ANOVA. For microarray cross-platform comparisons we used a *p* value of <0.05 and a 1.2-fold change cutoff. Raw data from these datasets were analyzed with Partek Genomics Suites under similar conditions used for the CGC data. In order to avoid cross-platform heterogeneity we focused only on datasets generated on the Affymetrix chips.

GSE1297 [59] (Fig. 1) is a dataset collected from nine postmortem normal and 22 Alzheimer’s disease patients with different degrees of severity, obtained from the Brain Bank of the Alzheimer’s disease Center of the University of Kentucky. Only seven severe cases (mean Braak stage 5.9, mean age 84 years old) were included in this analysis.

GSE48350 [60] (Fig. 1) is a dataset collected from postmortem human hippocampus tissue from 33 controls and 26 Alzheimer’s patients (Braak stage 5–6, mean age 79–90 years old) and obtained from the NIH/NIA Alzheimer’s Disease Research Center Brain Bank (Bethesda, MD, USA) and profiled on the Affymetrix Human Genome U133 Plus 2.0 Array.

GSE36980 [61] (Fig. 1) is a dataset from hippocampus gene expression of ten controls and seven Alzheimer patients (83 to 105 years old at Braak stages 5 to 6) selected from autopsy samples obtained from Hisajama, Japan residents and profiled on the Affymetrix Human Gene 1.0 ST Arrays.

GSE12679 [62] (Fig. 1) is a dataset from a laser capture microdissection of microvascular endothelial cells and neurons from human dorsolateral prefrontal cortex obtained at autopsy from 12 control individuals, obtained from the Stanley Medical Research Institute brain collection (Bethesda, MD, USA) and profiled on the Affymetrix Human Genome U133 Plus 2.0 Array.

GSE12293 [63] (Fig. 1) is a dataset from autopsy samples of human dorsolateral prefrontal cortex obtained from the Stanley Medical Research Institute brain collection (Bethesda, MD, USA). Neurons from six control subjects and endothelial cells from seven control subjects were isolated by laser-capture microdissection and profiled on the Affymetrix Human Genome U133 Plus 2.0 Array.

Detailed demographic information may be consulted in the selected references [59–63].

GSE46871 [64] (Fig. 1) is a dataset from hippocampal gene expression of Tg2576 mice, a rodent model of Alzheimer’s disease, comparing untreated controls and mice treated with the angiotensin converting enzyme inhibitor (ACEI) captopril.

## Results

### Global gene expression analysis

#### Candesartan prevents glutamate-induced upregulation or downregulation of multiple genes in primary neurons

Differential gene expression comparing results from glutamate-treated neurons with those of vehicle-treated neurons yielded over 1100 transcripts significantly up- or downregulated by glutamate (Additional file 2: Table S2). Differential gene expression shows over 800 transcripts (including microRNAs and noncoding RNAs) that are up- or downregulated when candesartan was added prior to glutamate, as compared with those exposed to glutamate only (Additional file 2: Table S2).

Candesartan completely prevented the glutamate-induced up- or downregulation of 501 of these transcripts (twofold or higher, *p* < 0.05; Additional file 2: Table S2). Interestingly, the comparison of neurons treated with candesartan to the control untreated neurons generated only a few genes with no functional annotation that may represent the system noise, confirming the fact that candesartan does not have any significant effect on normal neurons (Additional file 2: Table S2).

### Confirmation of microarray results by qPCR

#### Candesartan prevents glutamate-induced gene upregulation

**Fig. 2:**
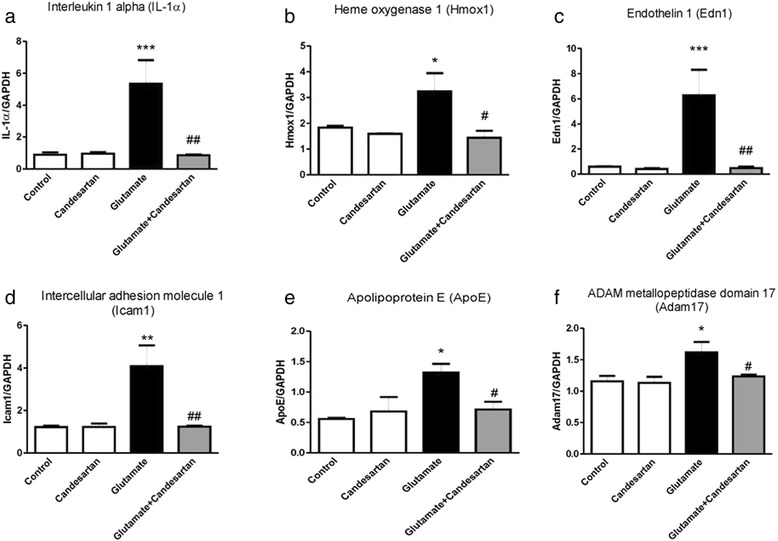
Candesartan prevents alterations in glutamate-induced gene expression in rat CGCs. Alterations in gene expression revealed by microarray analysis were confirmed by qPCR. Results are means ± SEM of at least three independent experiments. *p < 0.05, **p < 0.01, ***p < 0.001, glutamate vs. control; ^#^ *p* < 0.05, ^##^ *p* < 0.01, candesartan + glutamate vs glutamate. *GAPDH* glyceraldehyde-3-phosphate dehydrogenase. **a**: Interleukin 1 alpha. **b**: Heme oxygenase 1. **c**: Endothelin 1. **d**: Intercellular adhesion molecule 1.**e**: Apolipoprotein E. **f**: ADAM metallopeptidase domain 17

We confirmed microarray results by determination of expression of a number of genes upregulated by glutamate exposure by qPCR. In all these genes, candesartan pretreatment very significantly, and in many cases completely, prevented glutamate-induced upregulation. The exception was superoxide dismutase 2; in this case there was a clear trend, but the results were not statistically significant (Fig. 2; Additional file 3: Figure S1, Additional file 4: Figure S2 and Additional file 5: Figure S3).

### Pathway analysis

#### Specific diseases and functions, and upstream regulators associated with glutamate exposure and candesartan treatment

The list of functionally annotated genes from the glutamate versus glutamate + candesartan (423 unique genes) was submitted to IPA analysis [58]. As a result, cell movement, cell death and lesion formation, inflammation and synthesis of reactive oxygen species, diabetes and glucose metabolism, vascular disease and blood vessel development came top of the list of diseases and functions (Table 1; Additional file 6: Table S3). Many genes within these pathways were up- or downregulated by glutamate, and these changes were significantly prevented by candesartan (Additional file 2: Table S2).

**Table 1:** Representative IPA categories associated with glutamate andcandesartan-glutamate comparison

Categories	Diseases or functions annotation	*p* value	No. of molecules
Cellular movement	Cell movement	8.33E-52	170
Cell-to-cell signaling and interaction, cellular movement	Recruitment of cells	1.61E-49	72
Hematological system development and function, tissue morphology	Quantity of blood cells	4.80E-46	114
Inflammatory response	Inflammation of organ	1.61E-45	119
Organismal injury and abnormalities	Lesion formation	1.67E-44	73
Cardiovascular disease	Vascular disease	1.33E-42	105
Immunological disease	Systemic autoimmune syndrome	7.31E-41	108
Endocrine system disorders, gastrointestinal disease, metabolic disease	Diabetes mellitus	1.60E-36	102
Cell death and survival	Cell death	2.16E-35	191
Metabolic disease	Glucose metabolism disorder	1.13E-33	109
Inflammatory disease	Chronic inflammatory disorder	1.54E-33	94
Cardiovascular system development and function, organismal development	Development of blood vessel	1.07E-27	83
Free radical scavenging	Synthesis of reactive oxygen species	5.38E-27	59

The number of molecules represents the number of genes differentially expressed between glutamate and candesartan + glutamate that are part of the pathway category. The whole ingenuity pathway analysis (IPA) output is provided as Additional file 3: Table S3

Within the IPA program, analysis of upstream regulators of these differentially expressed genes included the well-known inflammatory associated cytokines tumor necrosis factor (TNF) alpha, IL-1β and interferon (IFN) gamma, LPS, the growth factor transforming growth factor (TGF) beta-1, and five upstream regulator drugs PD98059, SB203580, U0126, SP600125 and LY294002 (Table 2; Additional file 7: Table S4). Additional upstream regulators were amyloid precursor protein (APP), retinoid acid (Tretinoin) and apolipoprotein E (APOE) (Table 2; Additional file 7: Table S4).

**Table 2:** Representative top 30 upstream regulators of genes differentially expressed between glutamate and glutamate + candesartan

Upstream regulator	Molecule type	*p* value of overlap
Tumor necrosis factor	Cytokine	1.06E-74
Lipopolysaccharide	Chemical drug	3.26E-63
Interleukin-1beta	Cytokine	1.02E-61
Interferon gamma	Cytokine	4.75E-61
Transforming growth factor beta-1	Growth factor	1.99E-49
Nuclear factor kappa B (complex)	Complex	1.80E-43
Dexamethasone	Chemical drug	2.74E-42
Interleukin-6	Cytokine	2.16E-40
Colony Stimulating Factor 2 (CSF2)	Cytokine	1.57E-39
Interleukin-10	Cytokine	6.06E-39
Of Kappa Light Polypeptide Gene Cin B Cells (IKBKB)	Kinase	7.92E-39
Interleukin-13	Cytokine	1.46E-38
Poly rI:rC-RNA	Chemical reagent	4.53E-38
Signal transducer and activator of transcription 3 (STAT3)	Transcription regulator	1.80E-37
Amyloid precursor protein	Other	2.86E-37
Nuclear Factor Of Kappa Light Polypeptide Gene Enhancer in B Cell Inhibitor, Alpha (NFKBIA)	Transcription regulator	4.57E-36
Myeloid differentiation primary response gene 88 (MYD88)	Other	4.97E-36
Tretinoin	Chemical—endogenous mammalian	6.44E-36
*E. coli* B4 lipopolysaccharide	Chemical toxicant	1.36E-35
PD98059	Chemical—kinase inhibitor	1.85E-35
Beta-estradiol	Chemical—endogenous mammalian	9.64E-35
Interleukin-4	Cytokine	1.46E-34
Apolipoprotein E	Transporter	2.32E-33
Platelet-derived growth factor BB	Complex	2.99E-33
Conserved Helix -Loop Helix Ubiquitous Kinase (CHUK)	Kinase	6.05E-33
Toll-like receptor 4	Transmembrane receptor	2.55E-32
Angiotensinogen (AGT)	Growth factor	3.51E-32
Phorbol myristate acetate	Chemical drug	6.73E-32
SB203580	Chemical—kinase inhibitor	1.17E-31
Tumor necrosis factor superfamily member 11 (TNFSF11)	Cytokine	1.79E-31

A full and detailed list is provided as Additional file 7: Table S4

### Gene set enrichment analysis

#### Association with Alzheimer’s disease

**Fig. 3:**
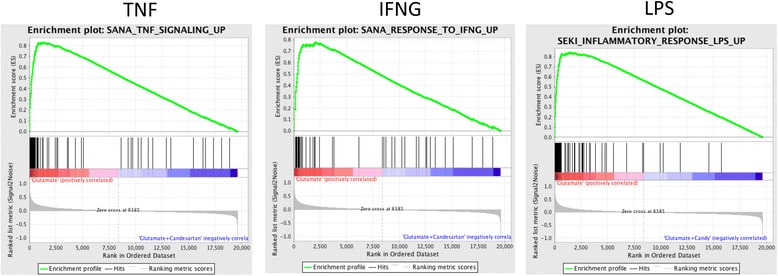
GSEA of ranked list of all genes comparing glutamate versus glutamate + candesartan. The figure includes a list of genes upregulated (*left*) or downregulated (*right*) in several gene sets associated with models of inflammation by interferon gamma (*IFNG*; *p* value = 0, FDR = 0, enrichment score = 0.78), lipopolysaccharide (*LPS*; *p* value = 0, FDR = 0, enrichment score = 0.84) and tumor necrosis factor (*TNF*; *p* value = 0, FDR = 0, enrichment score = 0.83). Genes upregulated by IFNG, LPS and TNF are also upregulated by glutamate with the highest enrichment score, *p* value and FDR (see Additional file 8: Table S5 and Additional file 9: Table S9 for more detail)

To further define candesartan molecular and functional pathways, we ran our glutamate versus glutamate + candesartan CGC microarray data through GSEA [52]. As predicted, inflammatory pathways associated with IFN-γ, TNFα, IL-1β and LPS, and several mechanisms of apoptosis were the most statistically relevant pathways (Fig. 3; Additional file 8: Table S5 and Additional file 9: Table S9). Genes upregulated by excitotoxic concentrations of glutamate are associated with genes upregulated by the IFN-γ, IL-6, IL-1β, TNFα and LPS-induced inflammation, involving chemokine signaling, focal adhesion, actin cytoskeleton, apoptosis by nutrient deprivation and extracellular matrix receptor interaction pathways. The glutamate-induced upregulation of these genes was normalized by candesartan treatment. Conversely, candesartan prevented the glutamate-induced downregulation of genes associated with neuronal function (cholinergic, dopamine and gamma-aminobutyric acid A receptors) (Additional file 8: Table S5).

**Fig. 4:**
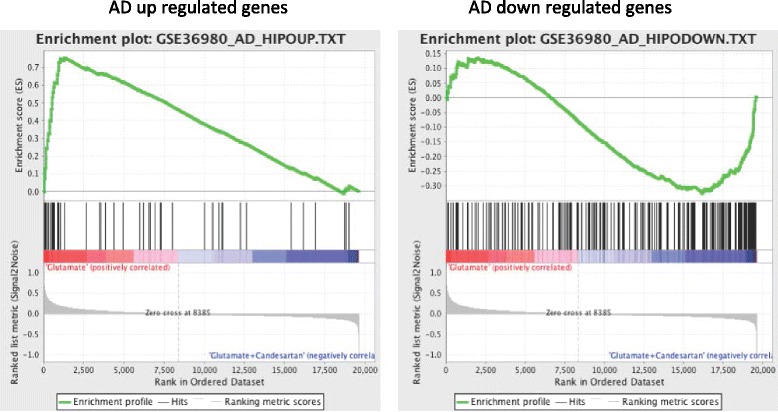
GSEA comparing the glutamate versus glutamate + candesartan genes with those from Alzheimer’s disease hippocampus. The figure is a ranked list of all genes comparing the glutamate versus glutamate + candesartan groups in comparison with a list of genes upregulated (*left*) or downregulated (*right*) in Alzheimer’s disease (*AD*) hippocampus depicted from the GEO dataset GSE36980. Genes upregulated in Alzheimer’s disease patients are upregulated by glutamate (*p* value = 0, FDR = 0, enrichment score = 0.75) and genes downregulated in Alzheimer’s disease patients are also downregulated by glutamate (*p* value = 0, FDR = 0.31, enrichment score = −0.32)

At the disease level, the GSEA shows a striking association with Alzheimer’s and Parkinson’s disease (Additional file 8: Table S5 and Additional file 9: Table S9). Genes that are upregulated in a published dataset (GSE36980 [61]; Fig. 1) of hippocampus samples obtained from Alzheimer’s disease patients strongly correlate with genes upregulated in neurons exposed to glutamate (*p* value = 0.0, FDR = 0.09, enrichment score = 0.75) (Fig. 4; Additional file 8: Table S5 and Additional file 9: Table S9). Conversely, genes that are downregulated in this published dataset strongly correlate with genes downregulated in neurons exposed to glutamate (Figs. 1 and 4; Additional file 8: Table S5 and Additional file 9: Table S9).

### Correlation of changes in gene expression in rat CGCs with those reported in a mouse model of Alzheimer’s disease

#### Association of candesartan treatment in CGCs and captopril treatment in APPswe mice

**Fig. 5:**
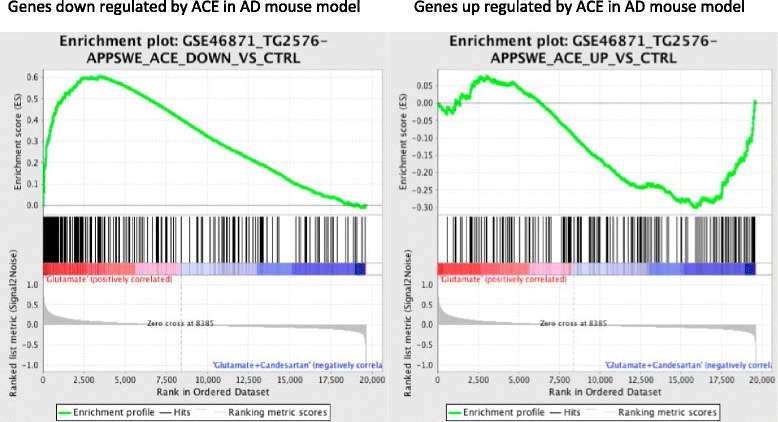
GSEA comparing the glutamate versus glutamate + candesartan genes with those from the APPswe mouse. The figure is a ranked list of all genes comparing the glutamate versus glutamate + candesartan groups in comparison with a list of genes upregulated (*left*) or downregulated (*right*) in the hippocampus of the APPswe mouse model of Alzheimer’s disease depicted from the GEO dataset GSE46871. Genes upregulated by glutamate in our neuronal culture are downregulated by treatment with an angiotensin converting enzyme (*ACE*) inhibitor in the APPswe mouse model (*p* value = 0, FDR = 0.0, enrichment score = −0.60). Inversely, genes downregulated by glutamate in our neuronal culture are upregulated by treatment with an ACE inhibitor in the APPswe mouse model (*p* value = 0, FDR = 0, enrichment score = −0.30)

Gene expression affected by glutamate and glutamate–candesartan in our model shows perfect correlation with the alterations in gene expression observed in the APPswe mouse model of Alzheimer’s disease treated with the ACEI captopril (GSEA 46871) [64]. Genes that were upregulated by glutamate in our study (effects prevented by candesartan) have been reported to be downregulated by captopril (*p* value = 0.0, FDR = 0.0, enrichment score = 0.60) and, inversely, genes downregulated by glutamate (this effect being prevented by candesartan in our model) were upregulated by captopril treatment in the APPswe mice (*p* value = 0.0, FDR = 0.0, enrichment score = −0.30) (Figs. 1 and 5).

### KEGG analysis

#### Association of differentially expressed genes in rat CGCs and Alzheimer’s disease hippocampus

We used the KEGG [53] Alzheimer’s disease pathways gene set to compare to genes differentially expressed in postmortem hippocampus from Alzheimer’s disease patients (GSEA 48350) [60] (Fig. 1) and their counterpart in our rat CGC study (Fig. 1). As seen in Table 3, within the KEGG Alzheimer’s disease 56 genes network, 23 of them are down regulated in hippocampus from Alzheimer’s disease patients (changes observed in GSE48350) and eight of them are also downregulated by glutamate in our rat neuronal study. Changes in seven of these eight genes are prevented by treatment with candesartan in our rat neuronal study. Moreover, of the 14 genes upregulated in hippocampus from Alzheimer’s patients, 12 were upregulated also by glutamate in our study, and in turn all these changes were prevented by candesartan treatment (Fig. 1 and Table 3).

**Table 3:** Look-up of Alzheimer’s disease associated genes from KEGG pathways and their expression in hippocampus from Alzheimer’s patients (GSE48350 dataset) and rat CGC glutamate + candesartan treatment

KEGG gene symbol	Gene symbol	GSE48350 hippocampus ctrl vs AD *p* value	GSE48350 hippocampus ctrl vs AD fold change	CGC glutamate vs ctrl *p* value	CGC glutamate vs ctrl fold change	CGC glutamate vs glutamate + candesartan *p* value	CGC glutamate vs glutamate + candesartan fold change
NMDAR	GRIN1	0.000	–2.184	0.037	–1.191	0.733	1.026
Cn	PPP3CB	0.000	–2.111	0.002	–1.245	0.001	1.277
CDK5	cdk5	0.000	–2.039	0.357	–1.041	0.436	1.035
p35	Cdk5r1	0.000	–2.031	0.774	1.040	0.391	–1.126
Cn	ppp3ca	0.000	–1.749	0.000	–1.221	0.003	1.158
SERCA	ATP2A2	0.000	–1.691	0.002	–1.176	0.001	1.183
PLC	PLCB1	0.000	–1.653	0.038	–1.060	0.000	1.147
ERK2	MAPK1	0.000	–1.578	0.012	–1.074	0.026	1.063
CxIII	uqcrfs1	0.000	–1.535	0.254	1.099	0.002	1.379
NOS	NOS1	0.000	–1.529	0.403	–1.060	0.38	1.063
SNCA	Snca	0.000	–1.472	0.755	1.044	0.242	1.179
GSK3B	GSK3B	0.000	–1.464	0.000	–1.236	0.000	1.167
CxV	Atp5a1	0.000	–1.407	0.011	1.143	0.023	1.123
TAU	mapt	0.000	–1.358	0.544	–1.051	0.899	1.01
CxI	Ndufv1	0.000	–1.348	0.090	1.128	0.002	1.285
VDCC	CACNA1C	0.007	–1.25	0.781	1.015	0.004	–1.209
CytC	COX4I1	0.000	–1.244	0.002	1.194	0.000	–1.237
Gq	GNAQ	0.001	–1.221	0.140	–1.054	0.029	–1.085
Fe65	APBB1	0.002	–1.195	0.376	1.127	0.765	–1.04
BID	BID	0.04	–1.192	0.008	1.116	0.561	–1.021
CxII	SDHA	0.002	–1.18	0.392	1.071	0.005	1.301
APP–BP1	Nae1	0.024	–1.145	0.021	–1.267	0.006	1.344
BAD	Bad	0.05	–1.143	0.396	–1.068	0.151	1.121
BACE	BACE1	0.224	–1.113	0.005	–1.302	0.003	1.326
PERK	eif2ak3	0.166	–1.108	0.001	–1.198	0.041	1.096
IRE1A	ERN1	0.229	–1.068	0.019	1.093	0.000	–1.284
CASP9	Casp9	0.245	–1.058	0.495	1.081	0.228	–1.15
PEN2	psenen	0.413	–1.052	0.006	–1.135	0.086	1.073
NEP	MME	0.497	–1.038	0.036	1.371	0.012	–1.485
IDE	IDE	0.898	1.006	0.074	–1.112	0.004	1.211
FADD	Fadd	0.716	1.015	0.067	1.146	0.001	–1.351
NCSTN	NCSTN	0.266	1.042	0.704	1.031	0.318	1.085
APH–1	aph1a	0.239	1.058	0.360	1.052	0.36	1.052
APH–1	APH1B	0.204	1.063	0.032	–1.121	0.336	–1.048
CASP12	CASP12	0.067	1.072	0.000	1.988	0.000	–1.742
ABAD	HSD17B10	0.181	1.081	0.000	1.351	0.049	–1.07
PLC	plcb3	0.018	1.085	0.000	1.652	0.000	–1.455
CASP3	Casp3	0.032	1.091	0.010	–1.234	0.02	1.204
LRP	lrp1	0.107	1.1	0.017	1.293	0.02	–1.283
APAF1	Apaf1	0.018	1.108	0.641	–1.034	0.458	1.055
PSEN	Psen1	0.011	1.117	0.096	–1.079	0.935	1.004
ATF6	ATF6B	0.07	1.14	0.001	1.248	0.01	–1.174
CALPAIN	CAPN1	0.003	1.154	0.000	1.490	0.000	–1.298
ADAM17	Adam17	0.000	1.165	0.000	2.442	0.000	–2.505
APOE	APOE	0.033	1.18	0.000	2.023	0.005	–1.325
RYR	RYR3	0.001	1.184	0.000	1.480	0.000	–1.707
CASP8	CASP8	0.008	1.242	0.002	1.180	0.01	–1.14
IP3R	ITPR1	0.024	1.271	0.002	1.245	0.031	–1.146
CASP7	casp7	0.001	1.28	0.000	1.139	0.000	–1.149
CaM	CALM2	0.001	1.305	0.004	1.219	0.006	–1.202
CaM	calml4	0.000	1.446	0.000	1.425	0.015	–1.226
LPL	Lpl	0.02	1.481	0.000	4.445	0.000	–2.672
FasTNFR1	TNFRSF1A	0.001	1.551	0.000	1.953	0.000	–1.615
APP	App	NA	NA	0.838	1.008	0.118	–1.07
GAPD	Gapdh	NA	NA	0.338	1.040	0.843	1.008
ERK1	Mapk3	NA	NA	0.040	1.084	0.006	–1.124

*AD* Alzheimer’s disease, *CGC* cerebellar granule cell, *ctrl* control, *KEGG* Kyoto encyclopedia of genes and genomes, *NA* not available

### Analysis of gene expression in specific cell populations

#### Differential alterations in gene expression preferentially expressed in neurons and cerebrovascular cells

We sought to define the predominant cellular origin of genes with altered expression in postmortem hippocampus from Alzheimer disease patients and their preferential expression in neurons or endothelial cells and the possible differential regulation of these genes by glutamate and glutamate–candesartan in our neuronal study. To this end we compared gene expression of two datasets generated from hippocampus of Alzheimer’s disease patients and normal controls (GSE48350 [60] and GSE36980 [61]) with two datasets generated from neurons and cerebrovascular endothelial cells from normal human dorsolateral prefrontal cortex, specifically isolated by laser capture microdissection (GSE12293 and GSE12679) [62, 63]. We then compared the genes predominantly expressed in neurons and cerebrovascular cells in the Alzheimer’s samples with genes regulated in our neuronal cultures by glutamate and by glutamate–candesartan.

**Fig. 6:**
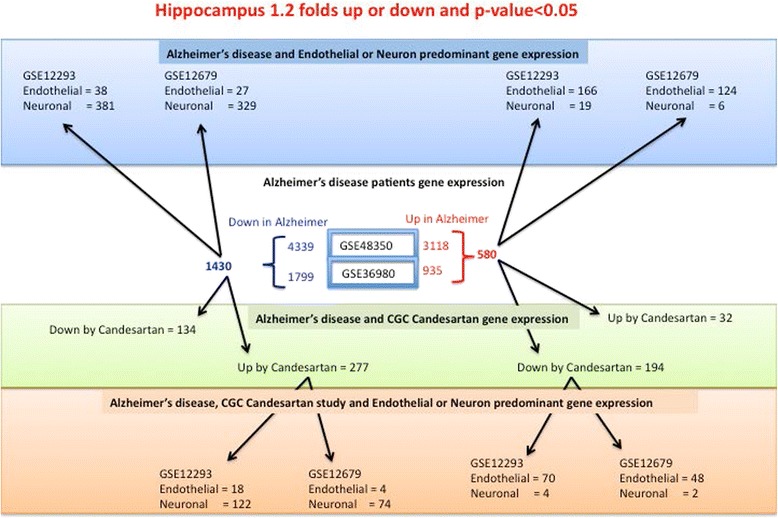
Cellular origin of gene expression in Alzheimer’s disease hippocampus. There are 580 genes upregulated (*red*) and 1430 downregulated (*blue*) differentially expressed transcripts in hippocampus from Alzheimer’s disease patients which are common to both GSE48350 and GSE1297 datasets. On the top side (*blue block*), the expression of these genes is then looked up in two datasets (GSE12293 and 12679) of normal laser capture microdissected neurons and cerebrovascular endothelial cells. On the bottom side (*green block*), the expression of these genes is compared to those differentially expressed in rat cerebellar granule cells (*CGCs*) treated with glutamate alone versus glutamate + candesartan and then looked-up in the two datasets (GSE12293 and 12679) of normal laser capture microdissected neurons and cerebrovascular endothelial cells (*orange block*). Note that the majority of the commonly expressed genes downregulated in Alzheimer’s disease hippocampus and upregulated by candesartan have a predominant expression in neurons. Conversely, the majority of the commonly expressed genes upregulated in Alzheimer’s disease hippocampus and downregulated by candesartan have a predominant endothelial expression

Using a 1.2-fold change cutoff and a *p* value below 0.05, we found 580 genes commonly up regulated in both Alzheimer patient datasets (Fig. 6). Of these 580 upregulated genes, 166 and 124 were predominantly expressed in cerebrovascular endothelial cells compared to 19 and 6 for neurons within GSE12293 [63] and GSE12679 [62] datasets, respectively. On the other hand, there are 1430 genes commonly downregulated in Alzheimer’s disease tissues. Of these, 381 and 329 were more expressed in neurons and only 38 and 27 were expressed in cerebrovascular endothelial cells within GSE12293 and GSE12679 datasets, respectively (Fig. 6).

We found striking correlations when we considered the preferential cellular expression, as revealed by the laser capture detection studies, with the gene expression in hippocampus from Alzheimer’s disease patients and with the results of our neuronal study. Most of the genes upregulated in Alzheimer’s disease hippocampus and by glutamate in our neuronal study that were normalized when candesartan was added to glutamate were of endothelial origin (70 versus 4 and 48 versus 2 in GSE12293 [63] and GSE12679 [62], respectively). Conversely, most of the genes downregulated in hippocampus from Alzheimer’s disease patients and by glutamate in our rat CGC study, changes normalized when candesartan was added to glutamate, were of neuronal origin (122 versus 18 and 74 versus 4 in GSE12293 [63] and GSE12679 [62], respectively) (Fig. 6).

For the genes upregulated in Alzheimer’s disease and by glutamate in our study, downregulated by candesartan, and, predominately expressed in endothelial cells, the pathway analysis revealed cellular movement/migration, extracellular matrix proteins, apoptosis, angiogenesis and vasculogenesis, and their most significant upstream regulators are beta-estradiol and TGFβ1 (Additional file 10: Figure S4, Additional file 11: Table S6 and Additional file 12: Table S7). On the other hand, genes upregulated in Alzheimer’s disease and downregulated by candesartan that are predominately expressed in neurons did not show any pathway significance, most probably due to the low number of genes.

Conversely, genes downregulated in Alzheimer’s disease and by glutamate in our neuronal study, which are upregulated by candesartan in our cultures, were predominately expressed in neurons. Pathway analysis revealed neurological diseases, neurodegeneration, neuronal apoptosis and disorders of basal ganglia (Additional file 13: Figure S5), and the most significant upstream regulator was the nuclear factor, erythroid 2-like 2 (NFE2L2, Nrf2) gene (Additional file 14: Table S8).

## Discussion

The overall goal of the study was to determine whether glutamate-induced alterations in gene expression in our primary neuronal culture were normalized by candesartan, and whether these changes correlated with alterations in gene expression in postmortem hippocampus of Alzheimer’s disease patients. We hypothesized that, if present, significant correlations would provide major preclinical evidence of beneficial therapeutic effects of candesartan.

There were several major findings in our study. Based on our results, we propose that candesartan may be neuroprotective on neuronal glutamate-induced injury. There were multiple functionally annotated genes strongly associated with Alzheimer’s disease and impressively correlated with alterations in gene expression in autopsy samples from Alzheimer’s disease hippocampus. We found novel functions differentially associated with genes predominantly expressed in neurons and in cerebrovascular endothelial cells.

Candesartan profoundly influenced glutamate-induced neuronal injury, since candesartan prevented glutamate-induced alterations in gene expression in about 800 of the over 1100 transcripts upregulated or downregulated by glutamate (Additional file 2: Table S2). Candesartan effects were unrelated to the proposed stimulation of angiotensin II (AT2) receptors by AT1R blockade, since AT2 receptors are not expressed in CGCs [25].

Using qPCR, we confirmed glutamate-induced upregulation, normalized by candesartan, of a number of these genes, including several factors with fundamental roles in APP metabolism and Alzheimer’s disease, such as ADAM metallopeptidase domain 17 [65–67] and APOE [68–72] (Fig. 2; Additional file 3: Figure S1, Additional file 4: Figure S2 and Additional file 5: Figure S3).

Inflammation plays a significant role in the pathogenesis of Alzheimer’s disease [6, 7, 73, 74]. Glutamate excitotoxicity upregulated many pro-inflammatory genes associated with Alzheimer’s disease [25, 75–89] and were normalized by candesartan (Fig. 2; Additional file 3: Figure S1, Additional file 4: Figure S4 and Additional file 5: Figure S3). Glutamate also upregulated the expression of some genes involved in anti-inflammatory processes, and candesartan prevented these changes (Fig. 2; Additional file 3: Figure S1, Additional file 4: Figure S2 and Additional file 5: Figure S3) [31, 90–93]. We hypothesize that while glutamate increases inflammation, at the same time it sets in motion a powerful anti-inflammatory response that is not necessary when the inflammatory response is prevented by candesartan.

Under the conditions of our experiments, we found that, when added after glutamate injury, candesartan does not protect neurons from cell injury [25]. We interpreted that candesartan administration, although it may not reverse glutamate-induced cell injury which has already occurred, will prevent further glutamate-induced injury. Since glutamate excitotoxicity is a long-term process during progression of Alzheimer’s disease [3, 8–10, 12], we believe our results are translationally relevant. The IPA analysis of the list of functionally annotated genes with their expression altered by glutamate and normalized when compared with the glutamate + candesartan group (over 400 genes) supported the proposed key role of inflammation in the pathogenesis of Alzheimer’s disease, [6, 7], agreed with the demonstrated major anti-inflammatory effect of candesartan [22, 27], and revealed many additional and novel diseases and functions, such as cell death and lesion formation, diabetes and glucose metabolism and vascular disease main risk factors for Alzheimer’s disease [4, 5] (Table 1; Additional file 6: Table S3).

Furthermore, IPA analysis of upstream regulators of these genes included APP, APOE and retinoic acid (Tretionin), which play major roles in Alzheimer’s disease [65–72] (Table 2; Additional file 7: Table S4) and revealed five kinase inhibitors, PD98059, SB203580, U0126, SP600125 and LY294002 (Table 2), that are part of the mitogen-activated protein kinase kinase/c-Jun N-terminal kinase/extracellular regulated kinase/p38-mitogen activated kinase/TGFβ-1 (MEK/JNK/ERK1/2/p38/TGFβ) pathways, reduce inflammation and toxicity, and have been associated with Alzheimer’s disease [85, 94–98]. We found that the influence of PD98059 and SB203580 over inflammatory genes was similar to that revealed by candesartan in our study [94, 95]. In support of the present findings, we have earlier reported that AT1R blockade prevents glutamate-induced ERK1/2, JNK and c-Jun activation [25, 29], demonstrating that the effect of candesartan is upstream of ERK1/2-p38MAPK.

GSEA supported the findings revealed by IPA. Inflammatory, chemokine signaling, focal adhesion, actin cytoskeleton, apoptosis and extracellular matrix receptor interaction pathways were most relevant, and the expression of the associated genes, upregulated by glutamate, was normalized by candesartan (Fig. 3; Additional file 8: Table S5). Conversely, candesartan prevented the glutamate-induced downregulation of genes associated with neuronal function (Additional file 8: Table S5).

Many genes (19 out of 53) in the KEGG Alzheimer’s disease reference pathway were altered in postmortem Alzheimer’s disease patients and by glutamate and normalized by candesartan. The pathways included mitochondrial dysfunction, APP processing, including β-secretase (BACE1), apoptosis, DNA damage, lipid peroxidase, Ca^2+^ signaling pathway and Ca^2+^ overload (Table 3).

Most remarkably, GSEA showed a striking association between changes observed in our neuronal culture and those observed in published datasets of hippocampal samples obtained from Alzheimer’s disease patients. Genes up- or downregulated in Alzheimer’s disease hippocampus [59–61] strongly correlated with genes up- or downregulated in neurons exposed to glutamate and prevented by candesartan (Fig. 4 and Table 3; Additional file 8: Table S5 and Additional file 9: Table S9).

Our results indicate that although the primary neurons studied here, CGCs, are not the primary targets for Alzheimer’s disease [44–46], upon glutamate injury they exhibited multiple mechanisms closely associated with those revealed in human hippocampal autopsy samples. Some of the glutamate-induced injury mechanisms observed in CGCs have been replicated in primary cortical neuronal cultures [25]. While analysis of postmortem samples has limitations because of the premortem agonal process and postmortem changes in glutamate metabolism, there was a striking correlation in alterations in gene expression between the two independent published datasets evaluated in our study. Furthermore, the normal controls used for the Alzheimer’s disease postmortem samples were also postmortem samples normalized for age and gender. Moreover, there were impressive correlations between our neuronal culture findings and those revealed in a mouse model of Alzheimer’s disease (Fig. 5), supporting the validity of our comparative analysis.

The predominant cellular expression of the genes altered in Alzheimer’s disease hippocampus and in our neuronal culture revealed two different pathological processes (Fig. 6). Multiple genes, upregulated in Alzheimer’s disease hippocampus and by glutamate in our neuronal culture, and normalized by candesartan, were predominantly expressed in human cerebrovascular endothelial cells when compared to neurons. IPA analysis of these genes revealed cellular movement/migration, extracellular matrix proteins, apoptosis, angiogenesis and vasculogenesis as principal functions controlled by these genes, and their most significant upstream regulators were TGFβ1 and beta-estradiol. TGFβ1 has been strongly associated with microvascular alterations in Alzheimer’s disease [99]. There is substantial evidence for a role of beta-estradiol, and in particular hippocampus-synthesized 17β-estradiol in synaptic plasticity and cognition [100, 101] and for neuroprotective effects of nonfeminizing estrogens [102]. The glutamate-induced upregulation of genes selectively overexpressed in cerebrovascular endothelial genes strongly supports the proposed role of alterations in the microvasculature in Alzheimer’s disease, not only as a risk factor but also playing a major role in its pathogenesis [4, 103–108].

Conversely, pathway analysis of genes predominantly expressed in human neurons when compared to human cerebrovascular endothelial cells and downregulated in Alzheimer’s disease hippocampus and by glutamate in our neuronal cultures, normalized by candesartan (Fig. 6), revealed neurological diseases, neurodegeneration, neuronal apoptosis and disorders of basal ganglia as principal related diseases. For these genes, the most significant upstream regulator was the NFE2L2 or Nrf2 gene that has been associated with the early stages of Alzheimer’s disease [109]. These results are concordant with the well-known loss of neural function in Alzheimer’s disease.

It is tempting to speculate that pathological processes in Alzheimer’s disease may be based on two sequential and/or concomitant processes: enhanced inflammation in microvascular endothelial cells and neuronal injury. Although candesartan is a drug that was designed to work on the hypertensive endothelial vascular system, our data indicates that candesartan may directly protect neurons from injury, a proposal supported by a previous observation [27].

Our report adds novel findings to the substantial body of evidence strongly suggesting that blockade of AT1R is a new avenue for the treatment of Alzheimer’s disease [22, 110]. Preclinical experiments indicate that excessive brain angiotensin II activity through overactivation of brain AT1R leads to cognitive loss associated with hippocampal long-term potentiation blockade, inhibition of the cholinergic system and stimulation of Aβ production and tau phosphorylation [22, 26, 111]. Of note, AT1R gene expression is upregulated by glutamate, and this change is normalized by candesartan (Additional file 2: Table S2).

Conversely, in preclinical models, AT1R blockade ameliorates hypertension, traumatic brain injury, brain ischemia and diabetes, the main modifying risk factors for Alzheimer’s disease, effects that include reduction of cognitive loss [22]. In addition, AT1R blockade ameliorates cognitive loss in most of the rodent models of Alzheimer’s disease by reducing brain inflammation, excessive oxidative stress and in some cases decreasing Aβ production, oligomerization, tau phosphorylation and reducing blood flow [22, 26, 31, 110–114].

Supporting the role of enhanced AT1R activity in Alzheimer’s disease, there was a correlation between alterations in gene expression in the APPswe mouse model of Alzheimer’s disease treated with captopril, an ACEI reducing angiotensin II formation, and those found in our study [64] (Fig. 5). ARBs reduce inflammation in human circulating monocytes exposed to LPS [28, 115], and prevent glutamate-induced neuronal apoptosis [25]. Clinical studies demonstrate that AT1R blockers reduce major risk factors for Alzheimer’s disease, [22, 110, 116]; observational and cohort studies reported that AT1R blockade delayed development of Alzheimer’s disease and protect cognition [22, 117]. There are increasing calls to conduct randomized controlled trials to effectively test the hypothesis that AT1R blockade may be a novel therapeutic approach for the treatment of Alzheimer’s disease [22, 117–119], and in particular including patients at the very early stages of the disease [120].

The mechanism of candesartan neuroprotection from glutamate excitotoxicity has been associated with blockade of the glutamate NMDA receptor [25]. In addition, candesartan neuroprotection may involve an increase in glutamate uptake into the cell [121]. Furthermore, AT1R blockade may not be the only mechanism responsible for the neuroprotective effect of candesartan. Some ARBs, in particular telmisartan and candesartan, are powerful activators of a major neuroprotective mechanism, the peroxisome proliferator-activated receptor gamma (PPARγ) [22, 25, 28, 33], and PPARγ activation plays a significant role in neuroprotection from glutamate excitotoxicity in cultured CGCs [25].

Our gene analysis revealed major associations of the gene alterations reported here with Parkinson’s disease, neurological diseases and neurodegeneration. These observations support the hypothesis that ARB neuroprotection may not only be effective in Alzheimer’s disease, but also in other neurodegenerative diseases [22, 23, 110].

## Conclusions

Our data may be interpreted as evidence of direct neuroprotective effects of candesartan in neurons, and of common disease processes that may underlie the in vitro acute gene alterations reported here and long-term mechanisms of cell injury in the late stages of Alzheimer’s disease. We provide novel evidence for candesartan neuroprotection through early molecular mechanisms of injury in this illness, such as glutamate-induced neuronal injury. Candesartan not only prevents inflammation but also novel pathogenic mechanisms such as risk factors for the disease and alterations in APP processing and mitochondrial function. The differential glutamate-induced alterations in genes preferentially expressed in cerebrovascular endothelial cells or neurons indicate the possibility of two different and interrelated pathogenic mechanisms, revealing multiple targets for candesartan neuroprotection. Our report supports the proposal to use ARBs as drugs of choice for the treatment of early cognitive loss.

## Additional files

Additional file 1: Table S1. List of primer sequences used for qPCR. (PDF 86 kb) Additional file 2: Table S2. Differentially expressed genes in glutamate and glutamate + candesartan groups. Differentially expressed genes between glutamate versus glutamate + candesartan (spreadsheet #2) and between glutamate versus control (spreadsheet #3) and between candesartan versus control (spreadsheet #4). The common genes (spreadsheet #1) represent genes that are differentially expressed in both comparisons. There is a complete reversal of the effect of glutamate by candesartan on all the 501 genes in common. (XLSX 267 kb) Additional file 3: Figure S1. Candesartan prevents glutamate-induced alterations in gene expression in rat CGCs. Alterations in gene expression revealed by microarray analysis were confirmed by qPCR. Results are means ± SEM of at least three independent experiments. **p* < 0.05, ***p* < 0.01, ****p* < 0.001, glutamate vs. control; ^#^ *p* < 0.05, ^##^ *p* < 0.01, ^###^ *p* < 00.1, Candesartan + glutamate vs glutamate. (PDF 74 kb) Additional file 4: Figure S2. Candesartan prevents glutamate-induced inflammation in rat CGCs. Alterations in gene expression revealed by microarray analysis were confirmed by qPCR. Results are means ± SEM of at least three independent experiments. **p* < 0.05, ***p* < 0.01, glutamate vs. control; ^#^ *p* < 0.05, ^##^ *p* < 0.01, candesartan + glutamate vs glutamate. (PDF 98 kb) Additional file 5: Figure S3. Candesartan prevents glutamate-induced inflammation in rat CGCs. Alterations in gene expression revealed by microarray analysis were confirmed by qPCR. Results are means ± SEM of at least three independent experiments. **p* < 0.05, ***p* < 0.01glutamate vs. control; ^#^ *p* < 0.05, ^##^ *p* < 0.01, candesartan + glutamate vs glutamate. (PDF 62 kb) Additional file 6: Table S3. IPA disease and/or function list for genes altered in glutamate versus glutamate + candesartan groups. (XLSX 67 kb) Additional file 7: Table S4. List of upstream regulation analysis for genes differentially expressed between glutamate and glutamate + candesartan. (XLSX 200 kb) Additional file 8: Table S5. GSEA for the genes altered in glutamate versus glutamate + candesartan. Table S5 is a GSEA for the genes with altered expression in our glutamate versus glutamate + candesartan groups, and their correlation with several gene sets/signatures associated with specific molecular functions, diseases, INFG, IL6, TNF, LPS and neuronal functions. (PDF 5309 kb) Additional file 9: Table S9. Complete GSEA analysis output of CGCs treated with glutamate and glutamate + candesartan and several inflammatory gene signatures and gene signature from postmortem Alzheimer’s disease patient and a mouse model of Alzheimer’s disease. (XLSX 4051 kb) Additional file 10: Figure S4. Pathways associated with genes preferentially expressed in cerebrovascular endothelial cells. Figure S4 notes pathways associated with genes that are upregulated in Alzheimer's disease and downregulated by candesartan in our neuronal cultures and are more expressed in endothelial cells. Genes are red in the outer circle and pathways are in orange in the inside circle. Orange dotted lines are for positive effect and blue dotted lines are for negative effect of the gene on the pathway. (PDF 940 kb) Additional file 11: Table S6. IPA of upstream regulators of genes preferentially expressed in cerebrovascular endothelial cells. Table S6 notes the upstream regulators of genes that are upregulated in Alzheimer’s disease and downregulated by candesartan in our neuronal cultures, and are of preferentially expressed in endothelial cells. (XLS 118 kb) Additional file 12: Table S7. IPA of diseases and functions annotation of genes preferentially expressed in cerebrovascular endothelial cells. Table S7 notes the diseases and functions that are upregulated in Alzheimer’s disease and downregulated by candesartan and are preferentially expressed in cerebrovascular endothelial cells. (XLS 126 kb) Additional file 13: Figure S5. Pathways associated with genes preferentially expressed in neurons. Figure S5 notes pathways that are downregulated in Alzheimer’s disease and upregulated by candesartan in our neuronal cultures. Genes are in green in the outer circle and pathways are in orange (activated) or blue (inhibited) in the inside circle. Orange dotted lines are for positive effect and blue dotted lines are for negative effect of the gene on the pathway. (PDF 892 kb) Additional file 14: Table S8. IPA of upstream regulators of genes preferentially expressed in neurons. Table S8 notes the upstream regulators of genes that are downregulated in Alzheimer’s disease and upregulated by candesartan in our neuronal cultures, and are preferentially expressed in human neurons. (XLS 40 kb)

## Abbreviations

Aβ: Amyloid beta
ACEI: Angiotensin converting enzyme inhibitor
ANOVA: Analysis of variance
APOE: Apolipoprotein E
APP: Amyloid precursor protein
ARB: Angiotensin receptor blocker
AT1R: Angiotensin 1 receptor
AT2: Angiotensin 2 receptor
cDNA: Complementary DNA
CGC: Cerebellar granule cell
ERK: Extracellular regulated kinase
GEO: Gene Expression Omnibus
GSEA: Gene set enrichment analysis
IFN: Interferon
IL: Interleukin
IPA: Ingenuity pathway analysis
JNK: c-Jun N-terminal kinase
KEGG: Kyoto encyclopedia of genes and genomes
LPS: Lipopolysaccharide
MEK: Mitogen-activated protein kinase kinase
MSigDB: Broad Molecular Signatures Database v5.0
PPARγ: peroxisome proliferator-activated receptor gamma
qPCR: Quantitative real-time polymerase chain reaction
RMA: Robust multichip analysis
TGF: Transforming growth factor
TNF: Tumor necrosis factor
