# Supplementary material for: An integrative genome-wide transcriptome reveals that candesartan is neuroprotective and a candidate therapeutic for Alzheimer’s disease
Source: Alzheimers Res Ther. 2016 Jan 28;8:5. doi: 10.1186/s13195-015-0167-5 (PMC4731966; doi:10.1186/s13195-015-0167-5)
Supplement: Additional file 1: Table S1. — List of primer sequences used for qPCR. (PDF 86 kb) [file 13195_2015_167_MOESM1_ESM.pdf]

Additional Table 1. List of primer sequences used for qPCR.

Gene	Primers: Forward	Primers: Reverse
**Pro-inflammatory**		
interleukin 1 alpha (IL-1α)	CCTCGTCCTAAGTCACTCGC	GGCTGGTTCCACTAGGCTTT
prostaglandin-endoperoxide synthase 2 (Ptgs2) (COX-2)	ATGCTACCATCTGGCTTCGG	TGGAACAGTCGCTCGTCATC
Vascular cell adhesion molecule 1 (Vcam1)	GTGAATCTGGTTGGGAGAGACA	AACGGAATCCCCAACCTGTG
Intercellular adhesion molecule 1 (Icam1)	GCCTGGGGTTGGAGACTAAC	CTGTCTTCCCCAATGTCGCT
Cd84 molecule (Cd84)	TCCACTCCCCTATGGACCAA	CATGGCTGTTGCACAGTGAC
chemokine (C-X-C motif) ligand 10 (Cxcl10)	TGAAAGCGGTGAGCCAAAGA	TAGCCGCACACTGGGTAAAG
Cd68 molecule (Cd68)	CGTTACCCGGAGACGACAAT	TCCTTGGTGGCCTACAGAGT
complement component 3a receptor 1 (C3ar1)	ATTCCCATCCCTGAATCGGC	AGCCTCCCGCACAAACTAAA
chemokine (C-X-C motif) ligand 16 (Cxcl16)	GAATCTGCTGGATGTCGGCT	GAGTGTAAGGGCCAAGGGTC
chemokine (C-X-C motif) ligand 5 (Cxcl5)	TCAAGCTGCTCCTTTCTCGG	GACTTCCACCTTGGGGCAAT
nuclear protein, transcriptional regulator, 1 (Nupr1)	CACACTTCCCAGCAACCTGT	GGTACGTCCTTTCCGACCTC
tumor necrosis factor, alpha-induced protein 2 (Tnfaip2)	AGCCAGGGACAGGCTACATA	GACAGGAGTTAACGGCCCAA
nuclear factor of kappa light polypeptide gene enhancer in B-cells 2, p49/p100 (Nfkpb2)	CCGTTGCTACGATCCAGGTT	TCACCAGATAGGGGCCATCA
endothelin 1 (End1)	TTTGAAGACCGCGCTGAGAT	TGCAAAACGAAGAGGACGGT
peptidase inhibitor, clade E (nexin, plasminogen activator inhibitor type 1), member 1 (Serpine1)	CGTCTTCCTCCACAGCCATT	GTTGGATTGTGCCGAACCAC
toll-like receptor 7 (Tlr7)	GAAACGCCATTGGCCAAACT	TCAAGCCGGTTGTTGGAGAA
ADAM metallopeptidase domain 17 (Adam17)	TCACATGTGCGTTGTCTCCA	GAACCTGGGGCTAAGCAGTT
apolipoprotein E (ApoE)	TTGTTTCGGAAGGAGCTGACTGG	TCCCAGGGTTGGTCGCTTTG
**Anti-inflammatory**		
interleukin 1 receptor antagonist (IL-1rn)	AGCCTCACCAACACACCAAA	GCCAGAGTGATCAGGCAGTT
heme oxygenase (decycling) 1 (Hmox1)	GAACTGTGGTCGGTAGAGGC	GGGGAAAGCAGTCATGGTCA
annexin A1 (Anxa1)	GGTGTGGATGAGGCAACCAT	CAAGGTTTCATCCAGGGGCT
superoxide dismutase 2, mitochondrial (Sod2)	GCTTGAATTGCTTGGACGCT	GCCCCAACACAGAGATGGAA
**Housekeeping**		
Glyceraldehyde-3-phosphate (GAPDH)	ATGACTCTACCCACGGCAAG	TGGAAGATGGTGATGGGTTT
