# Supplementary figures and images for: An integrative genome-wide transcriptome reveals that candesartan is neuroprotective and a candidate therapeutic for Alzheimer’s disease
Source: Alzheimers Res Ther. 2016 Jan 28;8:5. doi: 10.1186/s13195-015-0167-5 (PMC4731966; doi:10.1186/s13195-015-0167-5)

## Additional Figure 1

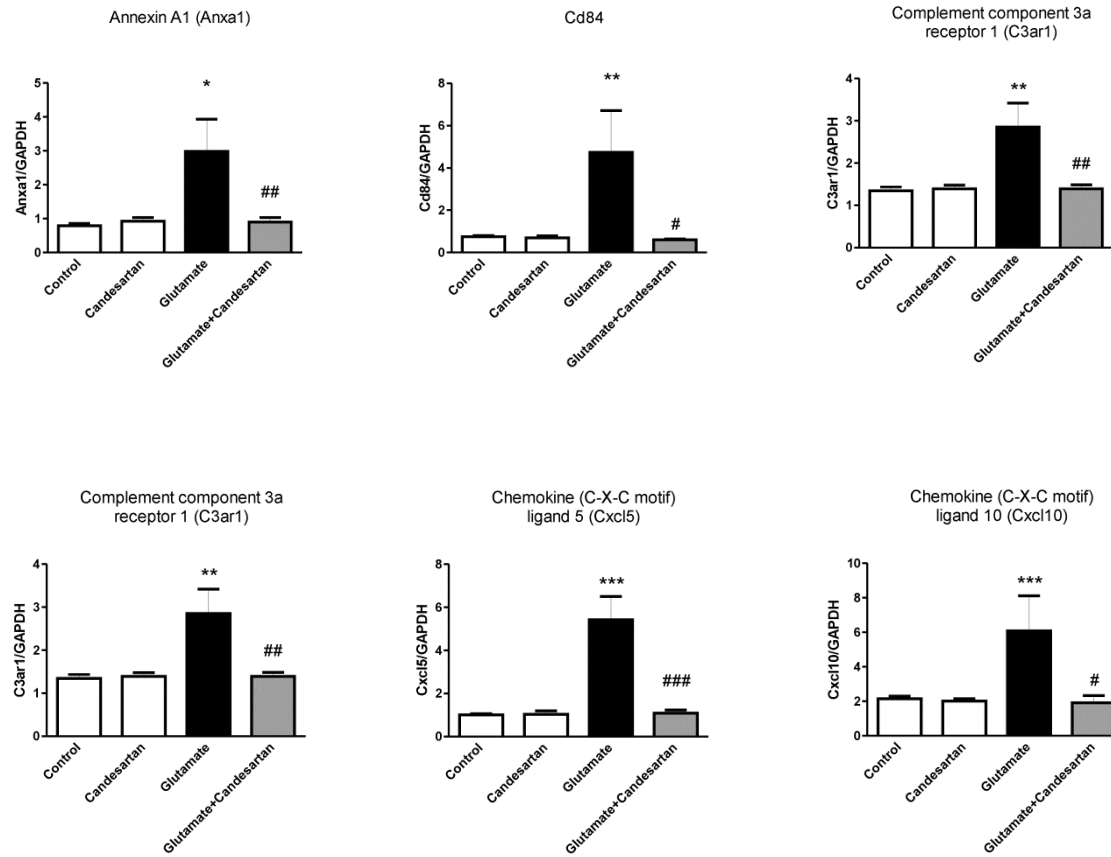

Supplement: Additional file 3: Figure S1. — Candesartan prevents glutamate-induced alterations in gene expression in rat CGCs. Alterations in gene expression revealed by microarray analysis were confirmed by qPCR. Results are means ± SEM of at least three independent experiments. *p < 0.05, **p < 0.01, ***p < 0.001, glutamate vs. control; # p < 0.05, ## p < 0.01, ### p < 00.1, Candesartan + glutamate vs glutamate. (PDF 74 kb) [file 13195_2015_167_MOESM3_ESM.pdf]

# Additional Figure 3

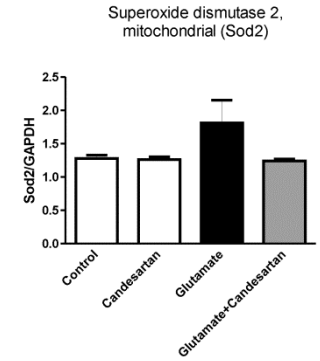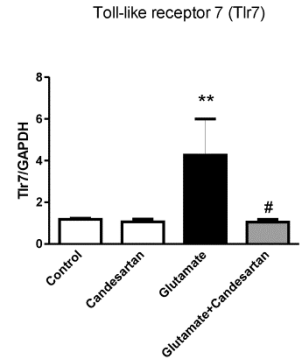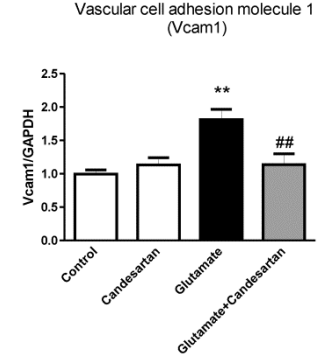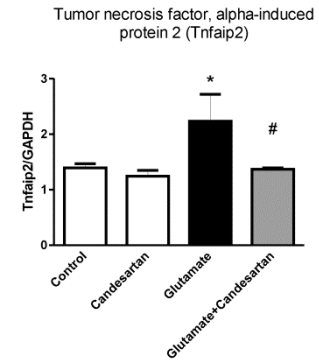

Supplement: Additional file 5: Figure S3. — Candesartan prevents glutamate-induced inflammation in rat CGCs. Alterations in gene expression revealed by microarray analysis were confirmed by qPCR. Results are means ± SEM of at least three independent experiments. *p < 0.05, **p < 0.01glutamate vs. control; # p < 0.05, ## p < 0.01, candesartan + glutamate vs glutamate. (PDF 62 kb) [file 13195_2015_167_MOESM5_ESM.pdf]

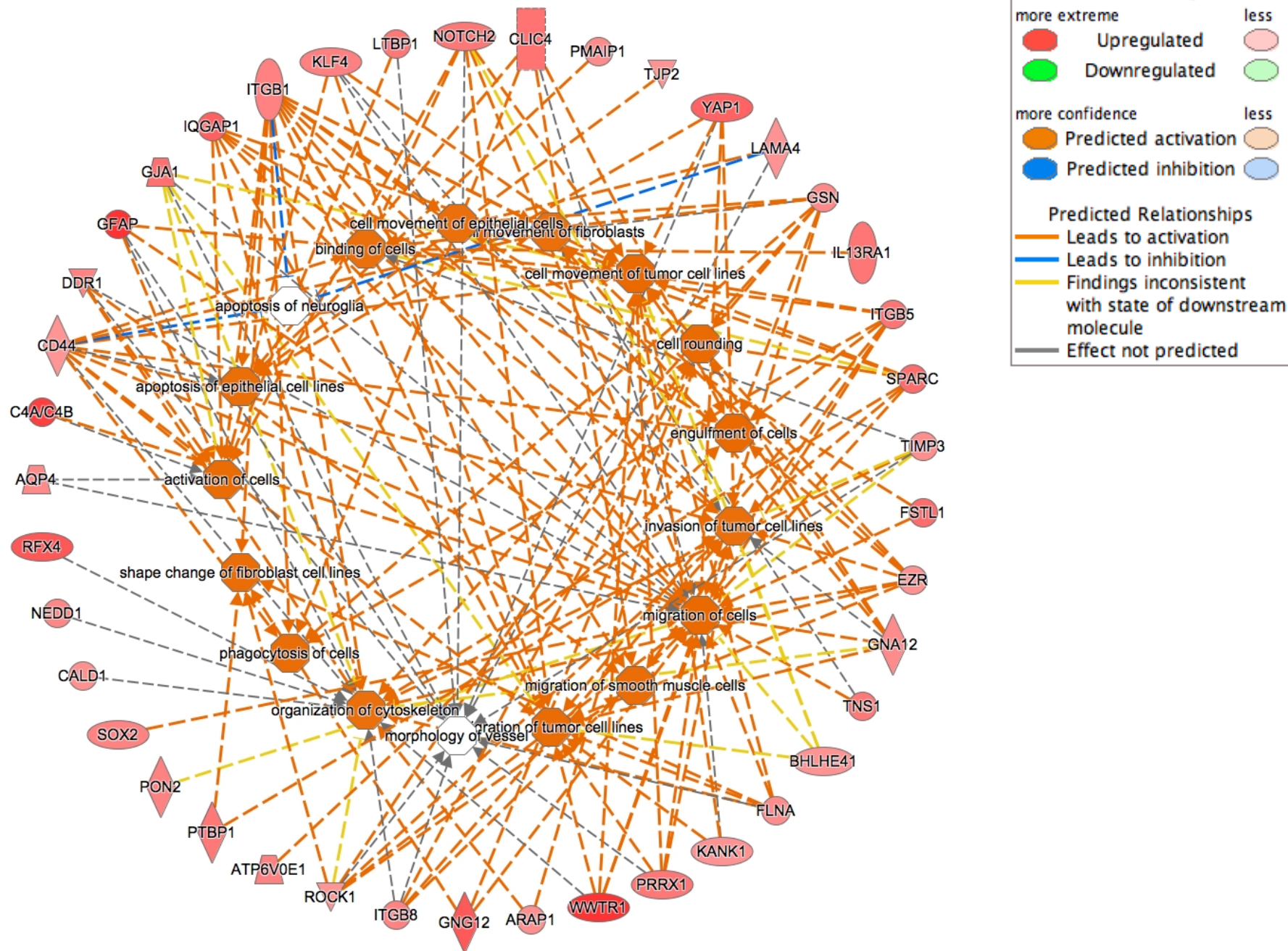

Supplement: Additional file 10: Figure S4. — Pathways associated with genes preferentially expressed in cerebrovascular endothelial cells. Figure S4 notes pathways associated with genes that are upregulated in Alzheimer's disease and downregulated by candesartan in our neuronal cultures and are more expressed in endothelial cells. Genes are red in the outer circle and pathways are in orange in the inside circle. Orange dotted lines are for positive effect and blue dotted lines are for negative effect of the gene on the pathway. (PDF 940 kb) [file 13195_2015_167_MOESM10_ESM.pdf]

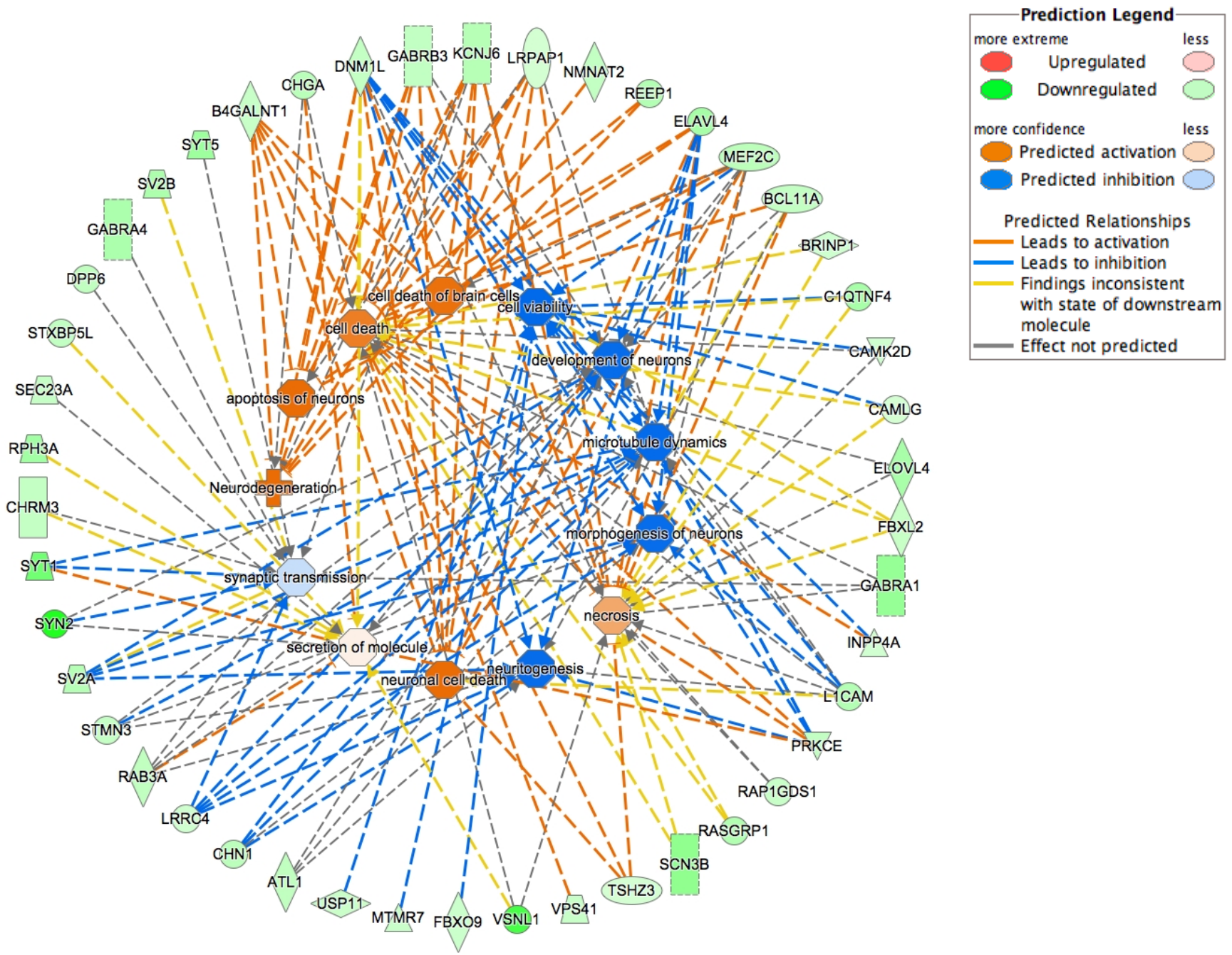

Supplement: Additional file 13: Figure S5. — Pathways associated with genes preferentially expressed in neurons. Figure S5 notes pathways that are downregulated in Alzheimer’s disease and upregulated by candesartan in our neuronal cultures. Genes are in green in the outer circle and pathways are in orange (activated) or blue (inhibited) in the inside circle. Orange dotted lines are for positive effect and blue dotted lines are for negative effect of the gene on the pathway. (PDF 892 kb) [file 13195_2015_167_MOESM13_ESM.pdf]
