# Supplementary material for: An integrative genome-wide transcriptome reveals that candesartan is neuroprotective and a candidate therapeutic for Alzheimer’s disease
Source: Alzheimers Res Ther. 2016 Jan 28;8:5. doi: 10.1186/s13195-015-0167-5 (PMC4731966; doi:10.1186/s13195-015-0167-5)

Additional Figure 2

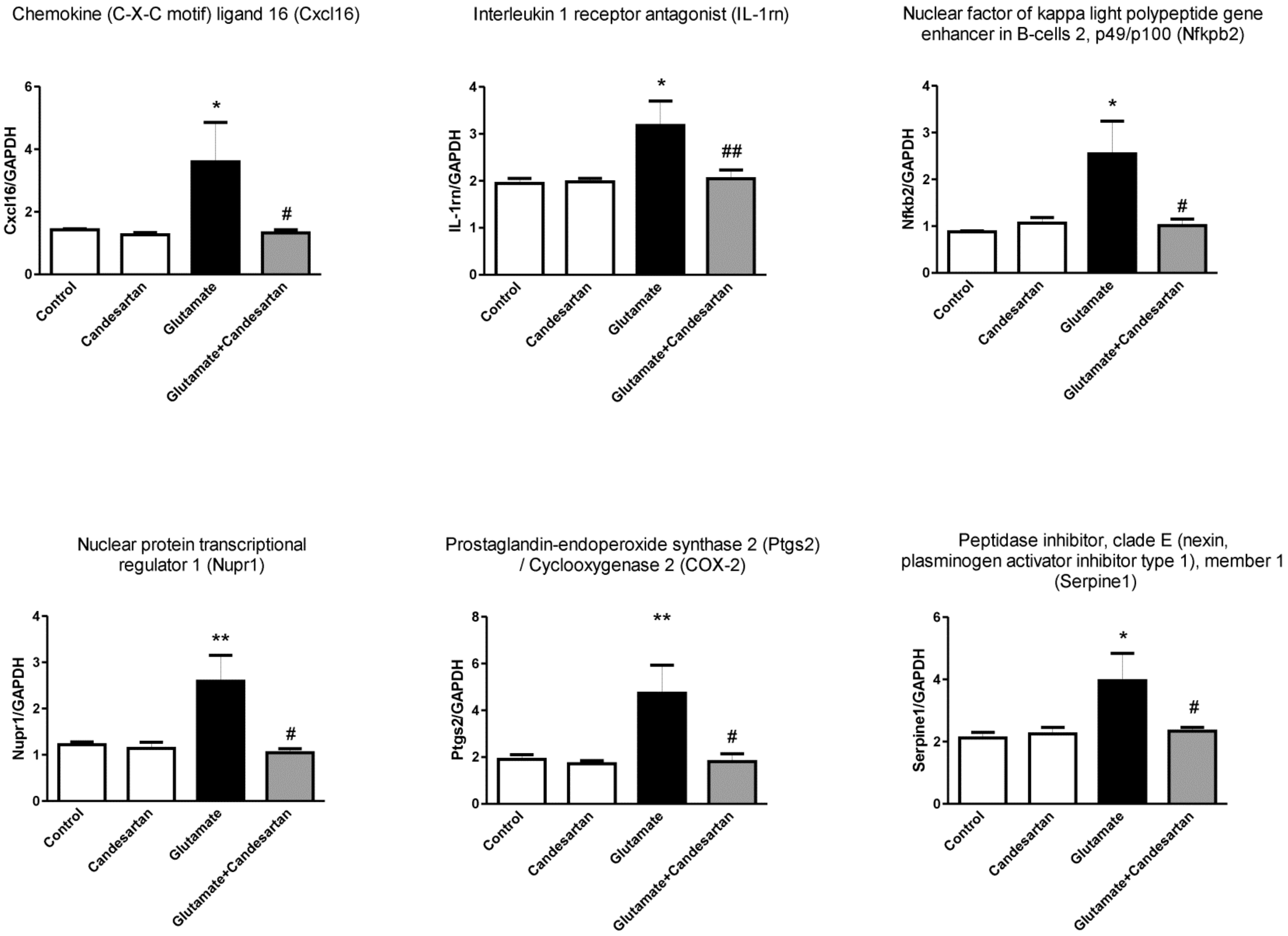

Supplement: Additional file 4: Figure S2. — Candesartan prevents glutamate-induced inflammation in rat CGCs. Alterations in gene expression revealed by microarray analysis were confirmed by qPCR. Results are means ± SEM of at least three independent experiments. *p < 0.05, **p < 0.01, glutamate vs. control; # p < 0.05, ## p < 0.01, candesartan + glutamate vs glutamate. (PDF 98 kb) [file 13195_2015_167_MOESM4_ESM.pdf]
