# Supplementary material for: An integrative genome-wide transcriptome reveals that candesartan is neuroprotective and a candidate therapeutic for Alzheimer’s disease
Source: Alzheimers Res Ther. 2016 Jan 28;8:5. doi: 10.1186/s13195-015-0167-5 (PMC4731966; doi:10.1186/s13195-015-0167-5)
Supplement: Additional file 8: Table S5. — GSEA for the genes altered in glutamate versus glutamate + candesartan. Table S5 is a GSEA for the genes with altered expression in our glutamate versus glutamate + candesartan groups, and their correlation with several gene sets/signatures associated with specific molecular functions, diseases, INFG, IL6, TNF, LPS and neuronal functions. (PDF 5309 kb) [file 13195_2015_167_MOESM8_ESM.pdf]

Gene Set enrichment of signatures associated with Alzheimer and Parkinson disease.

Table: GSEA Results Summary

Dataset	GlutaVSGlutaCandy_Anova_GSEA_Anova_GSEA.Glutamate_vs_Glutamate+Candesartan.cls #Glutamate_versus_Glutamate+Candesartan.Glutamate_vs_Glutamate+Candesartan.cls #Glutamate_versus_Glutamate+Candesartan_repos
Phenotype	Glutamate_vs_Glutamate+Candesartan.cls#Glutamate_versus_Glutamate+Candesartan_repos
Upregulated in class	Glutamate
GeneSet	GSE1297_ALZHEIMERUP
Enrichment Score (ES)	0.53333795
Normalized Enrichment Score (NES)	2.1286056
Nominal p-value	0.0
FDR q-value	0.0
FWER p-Value	0.0

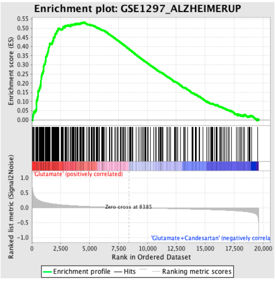

Fig 1: Enrichment plot: GSE1297_ALZHEIMERUP
Profile of the Running ES Score & Positions of GeneSet Members on the Rank Ordered List

Table: GSEA Results Summary

Dataset	GlutaVSGlutaCandy_Anova_GSEA_Anova_GSEA.Glutamate_vs_Glutamate+Candesartan.cls #Glutamate_versus_Glutamate+Candesartan.Glutamate_vs_Glutamate+Candesartan.cls #Glutamate_versus_Glutamate+Candesartan_repos
Phenotype	Glutamate_vs_Glutamate+Candesartan.cls#Glutamate_versus_Glutamate+Candesartan_repos
Upregulated in class	Glutamate
GeneSet	GSE36980_AD_HIPOUP.TXT
Enrichment Score (ES)	0.7571331
Normalized Enrichment Score (NES)	2.3819132
Nominal p-value	0.0
FDR q-value	0.0
FWER p-Value	0.0

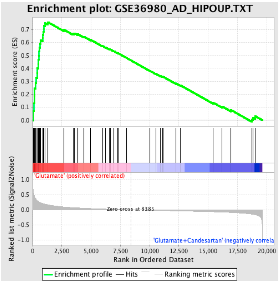

Fig 1: Enrichment plot: GSE36980_AD_HIPOUP.TXT
Profile of the Running ES Score & Positions of GeneSet Members on the Rank Ordered List

Table: GSEA Results Summary

Dataset	GlutaVSGlutaCandy_Anova_GSEA_Anova_GSEA.Glutamate_vs_Glutamate+Candesartan.cls #Glutamate_versus_Glutamate+Candesartan.Glutamate_vs_Glutamate+Candesartan.cls #Glutamate_versus_Glutamate+Candesartan_repos
Phenotype	Glutamate_vs_Glutamate+Candesartan.cls#Glutamate_versus_Glutamate+Candesartan_repos
Upregulated in class	Glutamate
GeneSet	GSE4757_AD_TANGLES_UP
Enrichment Score (ES)	0.72991675
Normalized Enrichment Score (NES)	2.421036
Nominal p-value	0.0
FDR q-value	0.0
FWER p-Value	0.0

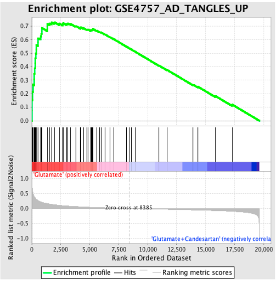

Fig 1: Enrichment plot: GSE4757_AD_TANGLES_UP
Profile of the Running ES Score & Positions of GeneSet Members on the Rank Ordered List

Table: GSEA Results Summary

Dataset	GlutaVSGlutaCandy_Anova_GSEA_Anova_GSEA.Glutamate_vs_Glutamate+Candesartan.cls #Glutamate_versus_Glutamate+Candesartan.Glutamate_vs_Glutamate+Candesartan.cls #Glutamate_versus_Glutamate+Candesartan_repos
Phenotype	Glutamate_vs_Glutamate+Candesartan.cls#Glutamate_versus_Glutamate+Candesartan_repos
Upregulated in class	Glutamate
GeneSet	GSE28146_ALZHEIMRSUP
Enrichment Score (ES)	0.48035675
Normalized Enrichment Score (NES)	1.7190361
Nominal p-value	0.0024509805
FDR q-value	0.002713607
FWER p-Value	0.026

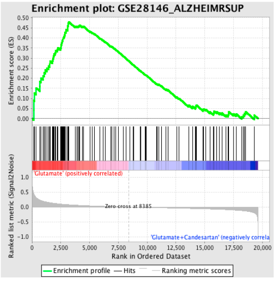

Fig 1: Enrichment plot: GSE28146_ALZHEIMRSUP
Profile of the Running ES Score & Positions of GeneSet Members on the Rank Ordered List

Table: GSEA Results Summary

Dataset	GlutaVSGlutaCandy_Anova_GSEA_Anova_GSEA.Glutamate_vs_Glutamate+Candesartan.cls #Glutamate_versus_Glutamate+Candesartan.Glutamate_vs_Glutamate+Candesartan.cls #Glutamate_versus_Glutamate+Candesartan_repos
Phenotype	Glutamate_vs_Glutamate+Candesartan.cls#Glutamate_versus_Glutamate+Candesartan_repos
Upregulated in class	Glutamate
GeneSet	GSE20292_PD-SN_UP
Enrichment Score (ES)	0.61630636
Normalized Enrichment Score (NES)	2.2405221
Nominal p-value	0.0
FDR q-value	0.0
FWER p-Value	0.0

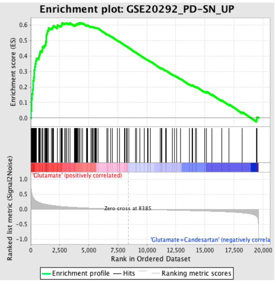

Fig 1: Enrichment plot: GSE20292_PD-SN_UP
Profile of the Running ES Score & Positions of GeneSet Members on the Rank Ordered List

Table: GSEA Results Summary

Dataset	GlutaVSGlutaCandy_Anova_GSEA_Anova_GSEA.Glutamate_vs_Glutamate+Candesartan.cls #Glutamate_versus_Glutamate+Candesartan.Glutamate_vs_Glutamate+Candesartan.cls #Glutamate_versus_Glutamate+Candesartan_repos
Phenotype	Glutamate_vs_Glutamate+Candesartan.cls#Glutamate_versus_Glutamate+Candesartan_repos
Upregulated in class	Glutamate
GeneSet	GSE7621_PD_SUBSTANCIANEGRA_UP
Enrichment Score (ES)	0.54610693
Normalized Enrichment Score (NES)	2.1333783
Nominal p-value	0.0
FDR q-value	0.0
FWER p-Value	0.0

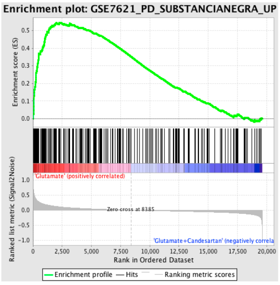

Fig 1: Enrichment plot: GSE7621_PD_SUBSTANCIANEGRA_UP
Profile of the Running ES Score & Positions of GeneSet Members on the Rank Ordered List

Table: GSEA Results Summary

Dataset	GlutaVSGlutaCandy_Anova_GSEA_Anova_GSEA.Glutamate_vs_Glutamate+Candesartan.cls #Glutamate_versus_Glutamate+Candesartan.Glutamate_vs_Glutamate+Candesartan.cls #Glutamate_versus_Glutamate+Candesartan_repos
Phenotype	Glutamate_vs_Glutamate+Candesartan.cls#Glutamate_versus_Glutamate+Candesartan_repos
Upregulated in class	Glutamate
GeneSet	GSE20291_PD_UP
Enrichment Score (ES)	0.6925817
Normalized Enrichment Score (NES)	1.8513502
Nominal p-value	0.0
FDR q-value	4.8534796E-4
FWER p-Value	0.0040

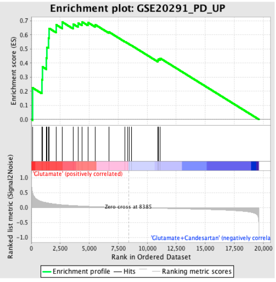

Fig 1: Enrichment plot: GSE20291_PD_UP
Profile of the Running ES Score & Positions of GeneSet Members on the Rank Ordered List

Table: GSEA Results Summary

Dataset	GlutaVSGlutaCandy_Anova_GSEA_Anova_GSEA.Glutamate_vs_Glutamate+Candesartan.cls #Glutamate_versus_Glutamate+Candesartan.Glutamate_vs_Glutamate+Candesartan.cls #Glutamate_versus_Glutamate+Candesartan_repos
Phenotype	Glutamate_vs_Glutamate+Candesartan.cls#Glutamate_versus_Glutamate+Candesartan_repos
Upregulated in class	Glutamate+Candesartan
GeneSet	GSE36980_AD_HIPODOWN.TXT
Enrichment Score (ES)	-0.3267033
Normalized Enrichment Score (NES)	-1.5589653
Nominal p-value	0.0
FDR q-value	0.031589475
FWER p-Value	0.048

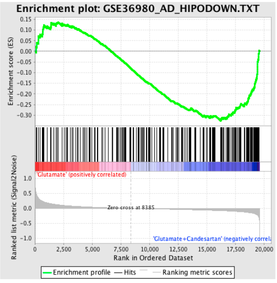

Fig 1: Enrichment plot: GSE36980_AD_HIPODOWN.TXT
Profile of the Running ES Score & Positions of GeneSet Members on the Rank Ordered List

Gene Set enrichment of some molecular functions

Table: GSEA Results Summary

Dataset	GlutaVSGlutaCandy_Anova_GSEA_Anova_GSEA.CGC_Glutamate_vs_CGC_Glutamate+Candesartan.cls #CGC_Glutamate_versus_CGC_Glutamate+Candesartan.CGC_Glutamate_vs_CGC_Glutamate+Candesartan.cls #CGC_Glutamate_versus_CGC_Glutamate+Candesartan_repos
Phenotype	CGC_Glutamate_vs_CGC_Glutamate+Candesartan.cls#CGC_Glutamate_versus_CGC_Glutamate+Candesartan_repos
Upregulated in class	CGC_Glutamate
GeneSet	KEGG_FOCAL_ADHESION
Enrichment Score (ES)	0.59562594
Normalized Enrichment Score (NES)	1.5168557
Nominal p-value	0.0
FDR q-value	0.031620555
FWER p-Value	0.0

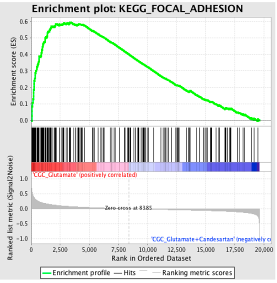

Fig 1: Enrichment plot: KEGG_FOCAL_ADHESION
Profile of the Running ES Score & Positions of GeneSet Members on the Rank Ordered List

Table: GSEA Results Summary

Dataset	GlutaVSGlutaCandy_Anova_GSEA_Anova_GSEA.CGC_Glutamate_vs_CGC_Glutamate+Candesartan.cls #CGC_Glutamate_versus_CGC_Glutamate+Candesartan.CGC_Glutamate_vs_CGC_Glutamate+Candesartan.cls #CGC_Glutamate_versus_CGC_Glutamate+Candesartan_repos
Phenotype	CGC_Glutamate_vs_CGC_Glutamate+Candesartan.cls#CGC_Glutamate_versus_CGC_Glutamate+Candesartan_repos
Upregulated in class	CGC_Glutamate
GeneSet	KEGG_CHEMOKINE_SIGNALING_PATHWAY
Enrichment Score (ES)	0.6631846
Normalized Enrichment Score (NES)	1.5347679
Nominal p-value	0.0
FDR q-value	0.027944112
FWER p-Value	0.0

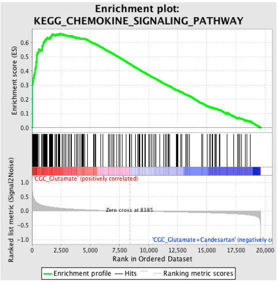

Fig 1: Enrichment plot: KEGG_CHEMOKINE_SIGNALING_PATHWAY
Profile of the Running ES Score & Positions of GeneSet Members on the Rank Ordered List

Table: GSEA Results Summary

Dataset	GlutaVSGlutaCandy_Anova_GSEA_Anova_GSEA.CGC_Glutamate_vs_CGC_Glutamate+Candesartan.cls #CGC_Glutamate_versus_CGC_Glutamate+Candesartan.CGC_Glutamate_vs_CGC_Glutamate+Candesartan.cls #CGC_Glutamate_versus_CGC_Glutamate+Candesartan_repos
Phenotype	CGC_Glutamate_vs_CGC_Glutamate+Candesartan.cls#CGC_Glutamate_versus_CGC_Glutamate+Candesartan_repos
Upregulated in class	CGC_Glutamate
GeneSet	KEGG_REGULATION_OF_ACTIN_CYTOSKELETON
Enrichment Score (ES)	0.50974125
Normalized Enrichment Score (NES)	1.618726
Nominal p-value	0.0
FDR q-value	0.23295964
FWER p-Value	0.278

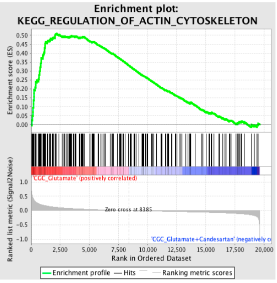

Fig 1: Enrichment plot: KEGG_REGULATION_OF_ACTIN_CYTOSKELETON
Profile of the Running ES Score & Positions of GeneSet Members on the Rank Ordered List

Table: GSEA Results Summary

Dataset	GlutaVSGlutaCandy_Anova_GSEA_Anova_GSEA.CGC_Glutamate_vs_CGC_Glutamate+Candesartan.cls #CGC_Glutamate_versus_CGC_Glutamate+Candesartan.CGC_Glutamate_vs_CGC_Glutamate+Candesartan.cls #CGC_Glutamate_versus_CGC_Glutamate+Candesartan_repos
Phenotype	CGC_Glutamate_vs_CGC_Glutamate+Candesartan.cls#CGC_Glutamate_versus_CGC_Glutamate+Candesartan_repos
Upregulated in class	CGC_Glutamate
GeneSet	APOPTOSIS_GO
Enrichment Score (ES)	0.5136034
Normalized Enrichment Score (NES)	1.5882267
Nominal p-value	0.0
FDR q-value	0.0861892
FWER p-Value	0.069

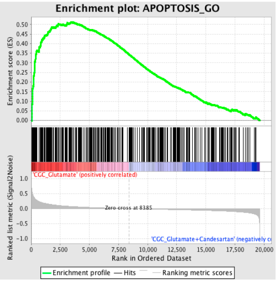

Fig 1: Enrichment plot: APOPTOSIS_GO
Profile of the Running ES Score & Positions of GeneSet Members on the Rank Ordered List

Table: GSEA Results Summary

Dataset	GlutaVSGlutaCandy_Anova_GSEA_Anova_GSEA.CGC_Glutamate_vs_CGC_Glutamate+Candesartan.cls #CGC_Glutamate_versus_CGC_Glutamate+Candesartan.CGC_Glutamate_vs_CGC_Glutamate+Candesartan.cls #CGC_Glutamate_versus_CGC_Glutamate+Candesartan_repos
Phenotype	CGC_Glutamate_vs_CGC_Glutamate+Candesartan.cls#CGC_Glutamate_versus_CGC_Glutamate+Candesartan_repos
Upregulated in class	CGC_Glutamate
GeneSet	GRAESSMANN_APOPTOSIS_BY_SERUM_DEPRIVATION_UP
Enrichment Score (ES)	0.56783265
Normalized Enrichment Score (NES)	1.5417799
Nominal p-value	0.0
FDR q-value	0.09623961
FWER p-Value	0.142

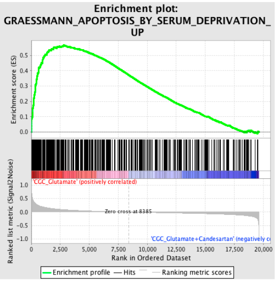

Fig 1: Enrichment plot: GRAESSMANN_APOPTOSIS_BY_SERUM_DEPRIVATION_UP
Profile of the Running ES Score & Positions of GeneSet Members on the Rank Ordered List

Table: GSEA Results Summary

Dataset	GlutaVSGlutaCandy_Anova_GSEA_Anova_GSEA.CGC_Glutamate_vs_CGC_Glutamate+Candesartan.cls #CGC_Glutamate_versus_CGC_Glutamate+Candesartan.CGC_Glutamate_vs_CGC_Glutamate+Candesartan.cls #CGC_Glutamate_versus_CGC_Glutamate+Candesartan_repos
Phenotype	CGC_Glutamate_vs_CGC_Glutamate+Candesartan.cls#CGC_Glutamate_versus_CGC_Glutamate+Candesartan_repos
Upregulated in class	CGC_Glutamate
GeneSet	CONCANNON_APOPTOSIS_BY_EPOXOMICIN_UP
Enrichment Score (ES)	0.60718966
Normalized Enrichment Score (NES)	1.5879139
Nominal p-value	0.0
FDR q-value	0.06456679
FWER p-Value	0.069

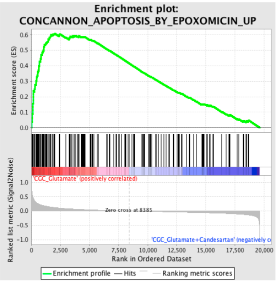

Fig 1: Enrichment plot: CONCANNON_APOPTOSIS_BY_EPOXOMICIN_UP
Profile of the Running ES Score & Positions of GeneSet Members on the Rank Ordered List

Table: GSEA Results Summary

Dataset	GlutaVSGlutaCandy_Anova_GSEA_Anova_GSEA.Glutamate_vs_Glutamate+Candesartan.cls #Glutamate_versus_Glutamate+Candesartan.Glutamate_vs_Glutamate+Candesartan.cls #Glutamate_versus_Glutamate+Candesartan_repos
Phenotype	Glutamate_vs_Glutamate+Candesartan.cls#Glutamate_versus_Glutamate+Candesartan_repos
Upregulated in class	Glutamate
GeneSet	KEGG_LYSOSOME
Enrichment Score (ES)	0.62195075
Normalized Enrichment Score (NES)	2.2002087
Nominal p-value	0.0
FDR q-value	0.0
FWER p-Value	0.0

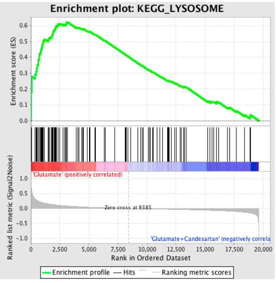

Fig 1: Enrichment plot: KEGG_LYSOSOME
Profile of the Running ES Score & Positions of GeneSet Members on the Rank Ordered List

Table: GSEA Results Summary

Dataset	GlutaVSGlutaCandy_Anova_GSEA_Anova_GSEA.CGC_Glutamate_vs_CGC_Glutamate+Candesartan.cls #CGC_Glutamate_versus_CGC_Glutamate+Candesartan.CGC_Glutamate_vs_CGC_Glutamate+Candesartan.cls #CGC_Glutamate_versus_CGC_Glutamate+Candesartan_repos
Phenotype	CGC_Glutamate_vs_CGC_Glutamate+Candesartan.cls#CGC_Glutamate_versus_CGC_Glutamate+Candesartan_repos
Upregulated in class	CGC_Glutamate
GeneSet	HOLLMANN_APOPTOSIS_VIA_CD40_UP
Enrichment Score (ES)	0.4453309
Normalized Enrichment Score (NES)	1.537235
Nominal p-value	0.0
FDR q-value	0.08573371
FWER p-Value	0.153

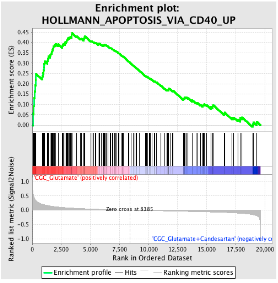

Fig 1: Enrichment plot: HOLLMANN_APOPTOSIS_VIA_CD40_UP
Profile of the Running ES Score & Positions of GeneSet Members on the Rank Ordered List

Table: GSEA Results Summary

Dataset	GlutaVSGlutaCandy_Anova_GSEA_Anova_GSEA.CGC_Glutamate_vs_CGC_Glutamate+Candesartan.cls #CGC_Glutamate_versus_CGC_Glutamate+Candesartan.CGC_Glutamate_vs_CGC_Glutamate+Candesartan.cls #CGC_Glutamate_versus_CGC_Glutamate+Candesartan_repos
Phenotype	CGC_Glutamate_vs_CGC_Glutamate+Candesartan.cls#CGC_Glutamate_versus_CGC_Glutamate+Candesartan_repos
Upregulated in class	CGC_Glutamate
GeneSet	REGULATION_OF_APOPTOSIS
Enrichment Score (ES)	0.50807893
Normalized Enrichment Score (NES)	1.6243286
Nominal p-value	0.0
FDR q-value	0.06936744
FWER p-Value	0.018

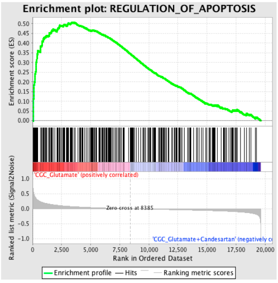

Fig 1: Enrichment plot: REGULATION_OF_APOPTOSIS
Profile of the Running ES Score & Positions of GeneSet Members on the Rank Ordered List

Table: GSEA Results Summary

Dataset	GlutaVSGlutaCandy_Anova_GSEA_Anova_GSEA.Glutamate_vs_Glutamate+Candesartan.cls #Glutamate_versus_Glutamate+Candesartan.Glutamate_vs_Glutamate+Candesartan.cls #Glutamate_versus_Glutamate+Candesartan_repos
Phenotype	Glutamate_vs_Glutamate+Candesartan.cls#Glutamate_versus_Glutamate+Candesartan_repos
Upregulated in class	Glutamate
GeneSet	KEGG_ECM_RECEPTOR_INTERACTION
Enrichment Score (ES)	0.612308
Normalized Enrichment Score (NES)	2.062227
Nominal p-value	0.0
FDR q-value	6.522718E-5
FWER p-Value	0.0010

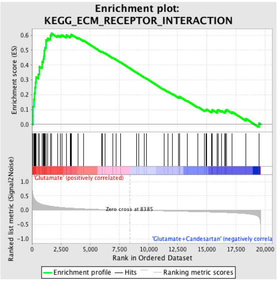

Fig 1: Enrichment plot: KEGG_ECM_RECEPTOR_INTERACTION
Profile of the Running ES Score & Positions of GeneSet Members on the Rank Ordered List

Table: GSEA Results Summary

Dataset	GlutaVSGlutaCandy_Anova_GSEA_Anova_GSEA.Glutamate_vs_Glutamate+Candesartan.cls #Glutamate_versus_Glutamate+Candesartan.Glutamate_vs_Glutamate+Candesartan.cls #Glutamate_versus_Glutamate+Candesartan_repos
Phenotype	Glutamate_vs_Glutamate+Candesartan.cls#Glutamate_versus_Glutamate+Candesartan_repos
Upregulated in class	Glutamate
GeneSet	LU_AGING_BRAIN_UP
Enrichment Score (ES)	0.62386596
Normalized Enrichment Score (NES)	2.3817763
Nominal p-value	0.0
FDR q-value	0.0
FWER p-Value	0.0

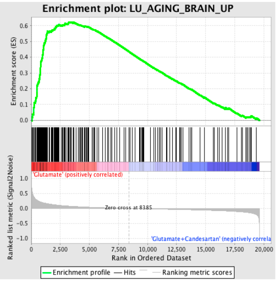

Fig 1: Enrichment plot: LU_AGING_BRAIN_UP
Profile of the Running ES Score & Positions of GeneSet Members on the Rank Ordered List

Gene Set enrichment of signatures associated with IFNG

Table: GSEA Results Summary

Dataset	GlutaVSGlutaCandy_Anova_GSEA_Anova_GSEA.Glutamate_vs_Glutamate+Candesartan.cls #Glutamate_versus_Glutamate+Candesartan.Glutamate_vs_Glutamate+Candesartan.cls #Glutamate_versus_Glutamate+Candesartan_repos
Phenotype	Glutamate_vs_Glutamate+Candesartan.cls#Glutamate_versus_Glutamate+Candesartan_repos
Upregulated in class	Glutamate
GeneSet	SANA_RESPONSE_TO_IFNG_UP
Enrichment Score (ES)	0.7806682
Normalized Enrichment Score (NES)	2.5007231
Nominal p-value	0.0
FDR q-value	0.0
FWER p-Value	0.0

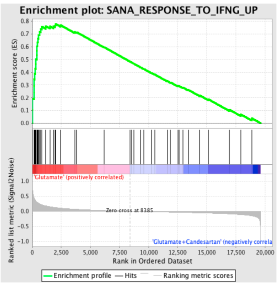

Fig 1: Enrichment plot: SANA_RESPONSE_TO_IFNG_UP
Profile of the Running ES Score & Positions of GeneSet Members on the Rank Ordered List

Table: GSEA Results Summary

Dataset	GlutaVSGlutaCandy_Anova_GSEA_Anova_GSEA.Glutamate_vs_Glutamate+Candesartan.cls #Glutamate_versus_Glutamate+Candesartan.Glutamate_vs_Glutamate+Candesartan.cls #Glutamate_versus_Glutamate+Candesartan_repos
Phenotype	Glutamate_vs_Glutamate+Candesartan.cls#Glutamate_versus_Glutamate+Candesartan_repos
Upregulated in class	Glutamate
GeneSet	GSE1432_CTRL_VS_IFNG_6H_MICROGLIA_DN
Enrichment Score (ES)	0.66946614
Normalized Enrichment Score (NES)	2.4826155
Nominal p-value	0.0
FDR q-value	0.0
FWER p-Value	0.0

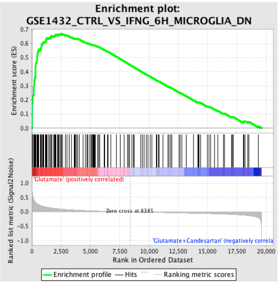

Fig 1: Enrichment plot: GSE1432_CTRL_VS_IFNG_6H_MICROGLIA_DN
Profile of the Running ES Score & Positions of GeneSet Members on the Rank Ordered List

Table: GSEA Results Summary

Dataset	GlutaVSGlutaCandy_Anova_GSEA_Anova_GSEA.Glutamate_vs_Glutamate+Candesartan.cls #Glutamate_versus_Glutamate+Candesartan.Glutamate_vs_Glutamate+Candesartan.cls #Glutamate_versus_Glutamate+Candesartan_repos
Phenotype	Glutamate_vs_Glutamate+Candesartan.cls#Glutamate_versus_Glutamate+Candesartan_repos
Upregulated in class	Glutamate
GeneSet	GSE1432_6H_VS_24H_IFNG_MICROGLIA_UP
Enrichment Score (ES)	0.6240706
Normalized Enrichment Score (NES)	2.3249002
Nominal p-value	0.0
FDR q-value	0.0
FWER p-Value	0.0

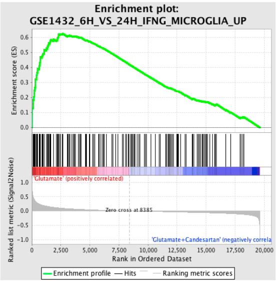

Fig 1: Enrichment plot: GSE1432_6H_VS_24H_IFNG_MICROGLIA_UP
Profile of the Running ES Score & Positions of GeneSet Members on the Rank Ordered List

Table: GSEA Results Summary

Dataset	GlutaVSGlutaCandy_Anova_GSEA_Anova_GSEA.Glutamate_vs_Glutamate+Candesartan.cls #Glutamate_versus_Glutamate+Candesartan.Glutamate_vs_Glutamate+Candesartan.cls #Glutamate_versus_Glutamate+Candesartan_repos
Phenotype	Glutamate_vs_Glutamate+Candesartan.cls#Glutamate_versus_Glutamate+Candesartan_repos
Upregulated in class	Glutamate
GeneSet	GSE3337_CTRL_VS_16H_IFNG_IN_CD8POS_DC_UP
Enrichment Score (ES)	0.58090687
Normalized Enrichment Score (NES)	2.183833
Nominal p-value	0.0
FDR q-value	0.0
FWER p-Value	0.0

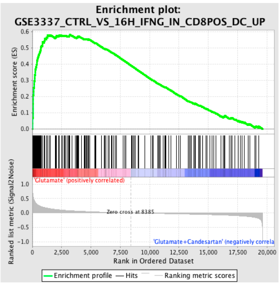

Fig 1: Enrichment plot: GSE3337_CTRL_VS_16H_IFNG_IN_CD8POS_DC_UP
Profile of the Running ES Score & Positions of GeneSet Members on the Rank Ordered List

Table: GSEA Results Summary

Dataset	GlutaVSGlutaCandy_Anova_GSEA_Anova_GSEA.Glutamate_vs_Glutamate+Candesartan.cls #Glutamate_versus_Glutamate+Candesartan.Glutamate_vs_Glutamate+Candesartan.cls #Glutamate_versus_Glutamate+Candesartan_repos
Phenotype	Glutamate_vs_Glutamate+Candesartan.cls#Glutamate_versus_Glutamate+Candesartan_repos
Upregulated in class	Glutamate
GeneSet	DASU_IL6_SIGNALING_UP
Enrichment Score (ES)	0.69930476
Normalized Enrichment Score (NES)	2.1907861
Nominal p-value	0.0
FDR q-value	0.0
FWER p-Value	0.0

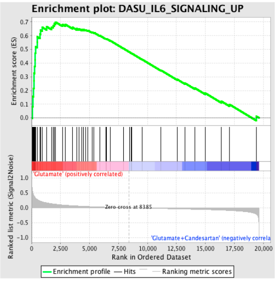

Fig 1: Enrichment plot: DASU_IL6_SIGNALING_UP
Profile of the Running ES Score & Positions of GeneSet Members on the Rank Ordered List

Gene Set enrichment of signatures associated with TNF

Table: GSEA Results Summary

Dataset	GlutaVSGlutaCandy_Anova_GSEA_Anova_GSEA.Glutamate_vs_Glutamate+Candesartan.cls #Glutamate_versus_Glutamate+Candesartan.Glutamate_vs_Glutamate+Candesartan.cls #Glutamate_versus_Glutamate+Candesartan_repos
Phenotype	Glutamate_vs_Glutamate+Candesartan.cls#Glutamate_versus_Glutamate+Candesartan_repos
Upregulated in class	Glutamate
GeneSet	SANA_TNF_SIGNALING_UP
Enrichment Score (ES)	0.8324428
Normalized Enrichment Score (NES)	2.7102234
Nominal p-value	0.0
FDR q-value	0.0
FWER p-Value	0.0

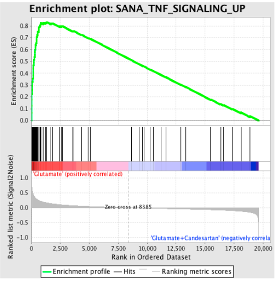

Fig 1: Enrichment plot: SANA_TNF_SIGNALING_UP
Profile of the Running ES Score & Positions of GeneSet Members on the Rank Ordered List

Table: GSEA Results Summary

Dataset	GlutaVSGlutaCandy_Anova_GSEA_Anova_GSEA.Glutamate_vs_Glutamate+Candesartan.cls #Glutamate_versus_Glutamate+Candesartan.Glutamate_vs_Glutamate+Candesartan.cls #Glutamate_versus_Glutamate+Candesartan_repos
Phenotype	Glutamate_vs_Glutamate+Candesartan.cls#Glutamate_versus_Glutamate+Candesartan_repos
Upregulated in class	Glutamate
GeneSet	ZHANG_RESPONSE_TO_IKK_INHIBITOR_AND_TNF_UP
Enrichment Score (ES)	0.72142255
Normalized Enrichment Score (NES)	2.6932478
Nominal p-value	0.0
FDR q-value	0.0
FWER p-Value	0.0

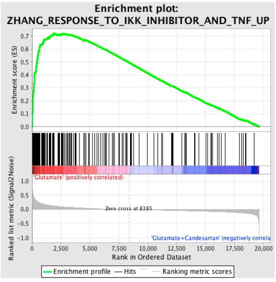

Fig 1: Enrichment plot: ZHANG_RESPONSE_TO_IKK_INHIBITOR_AND_TNF_UP
Profile of the Running ES Score & Positions of GeneSet Members on the Rank Ordered List

Table: GSEA Results Summary

Dataset	GlutaVSGlutaCandy_Anova_GSEA_Anova_GSEA.Glutamate_vs_Glutamate+Candesartan.cls #Glutamate_versus_Glutamate+Candesartan.Glutamate_vs_Glutamate+Candesartan.cls #Glutamate_versus_Glutamate+Candesartan_repos
Phenotype	Glutamate_vs_Glutamate+Candesartan.cls#Glutamate_versus_Glutamate+Candesartan_repos
Upregulated in class	Glutamate
GeneSet	PHONG_TNF_TARGETS_UP
Enrichment Score (ES)	0.8071003
Normalized Enrichment Score (NES)	2.6211133
Nominal p-value	0.0
FDR q-value	0.0
FWER p-Value	0.0

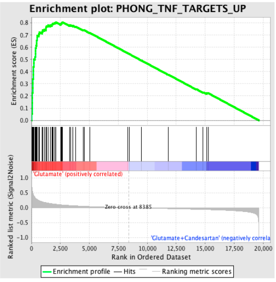

Fig 1: Enrichment plot: PHONG_TNF_TARGETS_UP
Profile of the Running ES Score & Positions of GeneSet Members on the Rank Ordered List

Table: GSEA Results Summary

Dataset	GlutaVSGlutaCandy_Anova_GSEA_Anova_GSEA.Glutamate_vs_Glutamate+Candesartan.cls #Glutamate_versus_Glutamate+Candesartan.Glutamate_vs_Glutamate+Candesartan.cls #Glutamate_versus_Glutamate+Candesartan_repos
Phenotype	Glutamate_vs_Glutamate+Candesartan.cls#Glutamate_versus_Glutamate+Candesartan_repos
Upregulated in class	Glutamate
GeneSet	GNF2_TNFRSF1B
Enrichment Score (ES)	0.81647867
Normalized Enrichment Score (NES)	2.5372636
Nominal p-value	0.0
FDR q-value	0.0
FWER p-Value	0.0

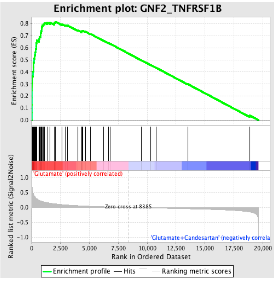

Fig 1: Enrichment plot: GNF2_TNFRSF1B
Profile of the Running ES Score & Positions of GeneSet Members on the Rank Ordered List

Table: GSEA Results Summary

Dataset	GlutaVSGlutaCandy_Anova_GSEA_Anova_GSEA.Glutamate_vs_Glutamate+Candesartan.cls #Glutamate_versus_Glutamate+Candesartan.Glutamate_vs_Glutamate+Candesartan.cls #Glutamate_versus_Glutamate+Candesartan_repos
Phenotype	Glutamate_vs_Glutamate+Candesartan.cls#Glutamate_versus_Glutamate+Candesartan_repos
Upregulated in class	Glutamate
GeneSet	PHONG_TNF_RESPONSE_VIA_P38_PARTIAL
Enrichment Score (ES)	0.6833173
Normalized Enrichment Score (NES)	2.5037653
Nominal p-value	0.0
FDR q-value	0.0
FWER p-Value	0.0

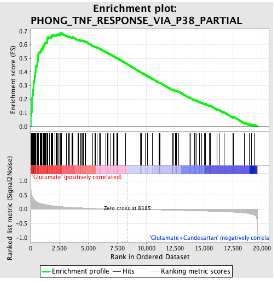

Fig 1: Enrichment plot: PHONG_TNF_RESPONSE_VIA_P38_PARTIAL
Profile of the Running ES Score & Positions of GeneSet Members on the Rank Ordered List

Table: GSEA Results Summary

Dataset	GlutaVSGlutaCandy_Anova_GSEA_Anova_GSEA.Glutamate_vs_Glutamate+Candesartan.cls #Glutamate_versus_Glutamate+Candesartan.Glutamate_vs_Glutamate+Candesartan.cls #Glutamate_versus_Glutamate+Candesartan_repos
Phenotype	Glutamate_vs_Glutamate+Candesartan.cls#Glutamate_versus_Glutamate+Candesartan_repos
Upregulated in class	Glutamate
GeneSet	PHONG_TNF_RESPONSE_NOT_VIA_P38
Enrichment Score (ES)	0.63340735
Normalized Enrichment Score (NES)	2.4844549
Nominal p-value	0.0
FDR q-value	0.0
FWER p-Value	0.0

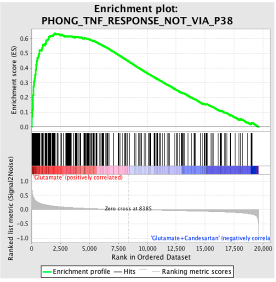

Fig 1: Enrichment plot: PHONG_TNF_RESPONSE_NOT_VIA_P38
Profile of the Running ES Score & Positions of GeneSet Members on the Rank Ordered List

Table: GSEA Results Summary

Dataset	GlutaVSGlutaCandy_Anova_GSEA_Anova_GSEA.Glutamate_vs_Glutamate+Candesartan.cls #Glutamate_versus_Glutamate+Candesartan.Glutamate_vs_Glutamate+Candesartan.cls #Glutamate_versus_Glutamate+Candesartan_repos
Phenotype	Glutamate_vs_Glutamate+Candesartan.cls#Glutamate_versus_Glutamate+Candesartan_repos
Upregulated in class	Glutamate
GeneSet	TIAN_TNF_SIGNALING_VIA_NFKB
Enrichment Score (ES)	0.89702123
Normalized Enrichment Score (NES)	2.445319
Nominal p-value	0.0
FDR q-value	0.0
FWER p-Value	0.0

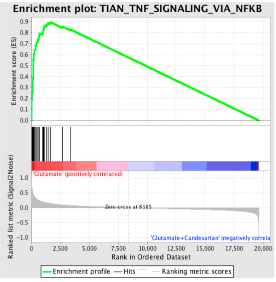

Fig 1: Enrichment plot: TIAN_TNF_SIGNALING_VIA_NFKB
Profile of the Running ES Score & Positions of GeneSet Members on the Rank Ordered List

Gene Set enrichment of signatures associated with LPS

Table: GSEA Results Summary

Dataset	GlutaVSGlutaCandy_Anova_GSEA_Anova_GSEA.Glutamate_vs_Glutamate+Candesartan.cls #Glutamate_versus_Glutamate+Candesartan.Glutamate_vs_Glutamate+Candesartan.cls #Glutamate_versus_Glutamate+Candesartan_repos
Phenotype	Glutamate_vs_Glutamate+Candesartan.cls#Glutamate_versus_Glutamate+Candesartan_repos
Upregulated in class	Glutamate
GeneSet	SEKI_INFLAMMATORY_RESPONSE_LPS_UP
Enrichment Score (ES)	0.8419115
Normalized Enrichment Score (NES)	2.8457441
Nominal p-value	0.0
FDR q-value	0.0
FWER p-Value	0.0

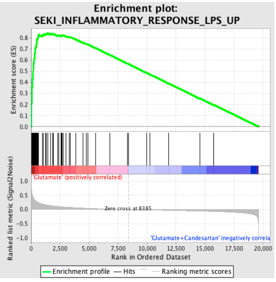

Fig 1: Enrichment plot: SEKI_INFLAMMATORY_RESPONSE_LPS_UP
Profile of the Running ES Score & Positions of GeneSet Members on the Rank Ordered List

Table: GSEA Results Summary

Dataset	GlutaVSGlutaCandy_Anova_GSEA_Anova_GSEA.Glutamate_vs_Glutamate+Candesartan.cls #Glutamate_versus_Glutamate+Candesartan.Glutamate_vs_Glutamate+Candesartan.cls #Glutamate_versus_Glutamate+Candesartan_repos
Phenotype	Glutamate_vs_Glutamate+Candesartan.cls#Glutamate_versus_Glutamate+Candesartan_repos
Upregulated in class	Glutamate
GeneSet	NEMETH_INFLAMMATORY_RESPONSE_LPS_UP
Enrichment Score (ES)	0.81028533
Normalized Enrichment Score (NES)	2.751519
Nominal p-value	0.0
FDR q-value	0.0
FWER p-Value	0.0

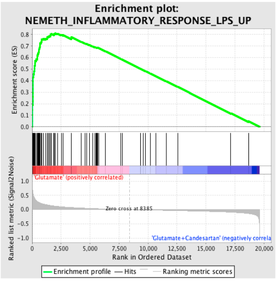

Fig 1: Enrichment plot: NEMETH_INFLAMMATORY_RESPONSE_LPS_UP
Profile of the Running ES Score & Positions of GeneSet Members on the Rank Ordered List

Table: GSEA Results Summary

Dataset	GlutaVSGlutaCandy_Anova_GSEA_Anova_GSEA.Glutamate_vs_Glutamate+Candesartan.cls #Glutamate_versus_Glutamate+Candesartan.Glutamate_vs_Glutamate+Candesartan.cls #Glutamate_versus_Glutamate+Candesartan_repos
Phenotype	Glutamate_vs_Glutamate+Candesartan.cls#Glutamate_versus_Glutamate+Candesartan_repos
Upregulated in class	Glutamate
GeneSet	GSE9988_LPS_VS_VEHICLE_TREATED_MONOCYTE_UP
Enrichment Score (ES)	0.7410895
Normalized Enrichment Score (NES)	2.7399554
Nominal p-value	0.0
FDR q-value	0.0
FWER p-Value	0.0

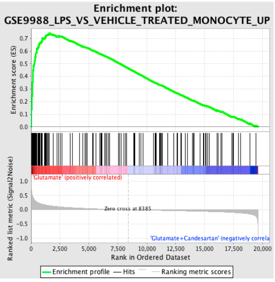

Fig 1: Enrichment plot: GSE9988_LPS_VS_VEHICLE_TREATED_MONOCYTE_UP
Profile of the Running ES Score & Positions of GeneSet Members on the Rank Ordered List

Table: GSEA Results Summary

Dataset	GlutaVSGlutaCandy_Anova_GSEA_Anova_GSEA.Glutamate_vs_Glutamate+Candesartan.cls #Glutamate_versus_Glutamate+Candesartan.Glutamate_vs_Glutamate+Candesartan.cls #Glutamate_versus_Glutamate+Candesartan_repos
Phenotype	Glutamate_vs_Glutamate+Candesartan.cls#Glutamate_versus_Glutamate+Candesartan_repos
Upregulated in class	Glutamate
GeneSet	MCBRYAN_PUBERTAL_TGFB1_TARGETS_UP
Enrichment Score (ES)	0.6393411
Normalized Enrichment Score (NES)	2.3786714
Nominal p-value	0.0
FDR q-value	0.0
FWER p-Value	0.0

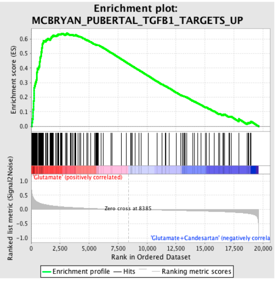

Fig 1: Enrichment plot: MCBRYAN_PUBERTAL_TGFB1_TARGETS_UP
Profile of the Running ES Score & Positions of GeneSet Members on the Rank Ordered List

Table: GSEA Results Summary

Dataset	GlutaVSGlutaCandy_Anova_GSEA_Anova_GSEA.Glutamate_vs_Glutamate+Candesartan.cls #Glutamate_versus_Glutamate+Candesartan.Glutamate_vs_Glutamate+Candesartan.cls #Glutamate_versus_Glutamate+Candesartan_repos
Phenotype	Glutamate_vs_Glutamate+Candesartan.cls#Glutamate_versus_Glutamate+Candesartan_repos
Upregulated in class	Glutamate
GeneSet	COULOUARN_TEMPORAL_TGFB1_SIGNATURE_UP
Enrichment Score (ES)	0.6850428
Normalized Enrichment Score (NES)	2.3426886
Nominal p-value	0.0
FDR q-value	0.0
FWER p-Value	0.0

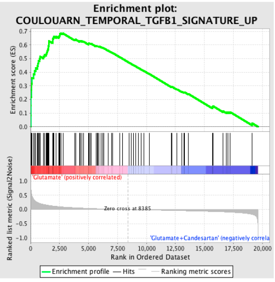

Fig 1: Enrichment plot: COULOUARN_TEMPORAL_TGFB1_SIGNATURE_UP
Profile of the Running ES Score & Positions of GeneSet Members on the Rank Ordered List

Table: GSEA Results Summary

Dataset	GlutaVSGlutaCandy_Anova_GSEA_Anova_GSEA.Glutamate_vs_Glutamate+Candesartan.cls #Glutamate_versus_Glutamate+Candesartan.Glutamate_vs_Glutamate+Candesartan.cls #Glutamate_versus_Glutamate+Candesartan_repos
Phenotype	Glutamate_vs_Glutamate+Candesartan.cls#Glutamate_versus_Glutamate+Candesartan_repos
Upregulated in class	Glutamate
GeneSet	PLASARI_TGFB1_TARGETS_10HR_UP
Enrichment Score (ES)	0.70949405
Normalized Enrichment Score (NES)	2.680638
Nominal p-value	0.0
FDR q-value	0.0
FWER p-Value	0.0

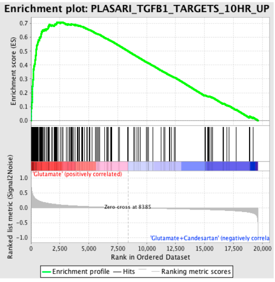

Fig 1: Enrichment plot: PLASARI_TGFB1_TARGETS_10HR_UP
Profile of the Running ES Score & Positions of GeneSet Members on the Rank Ordered List

Table: GSEA Results Summary

Dataset	GlutaVSGlutaCandy_Anova_GSEA_Anova_GSEA.Glutamate_vs_Glutamate+Candesartan.cls #Glutamate_versus_Glutamate+Candesartan.Glutamate_vs_Glutamate+Candesartan.cls #Glutamate_versus_Glutamate+Candesartan_repos
Phenotype	Glutamate_vs_Glutamate+Candesartan.cls#Glutamate_versus_Glutamate+Candesartan_repos
Upregulated in class	Glutamate
GeneSet	LABBE_TGFB1_TARGETS_UP
Enrichment Score (ES)	0.6738278
Normalized Enrichment Score (NES)	2.3622215
Nominal p-value	0.0
FDR q-value	0.0
FWER p-Value	0.0

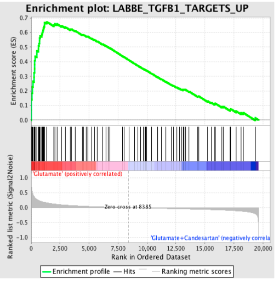

Fig 1: Enrichment plot: LABBE_TGFB1_TARGETS_UP
Profile of the Running ES Score & Positions of GeneSet Members on the Rank Ordered List

Table: GSEA Results Summary

Dataset	GlutaVSGlutaCandy_Anova_GSEA_Anova_GSEA.Glutamate_vs_Glutamate+Candesartan.cls #Glutamate_versus_Glutamate+Candesartan.Glutamate_vs_Glutamate+Candesartan.cls #Glutamate_versus_Glutamate+Candesartan_repos
Phenotype	Glutamate_vs_Glutamate+Candesartan.cls#Glutamate_versus_Glutamate+Candesartan_repos
Upregulated in class	Glutamate
GeneSet	VERRECCHIA_EARLY_RESPONSE_TO_TGFB1
Enrichment Score (ES)	0.7186787
Normalized Enrichment Score (NES)	2.3126495
Nominal p-value	0.0
FDR q-value	0.0
FWER p-Value	0.0

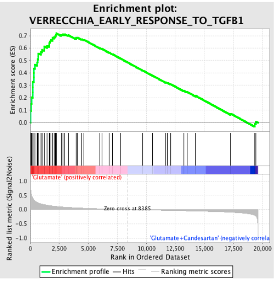

Fig 1: Enrichment plot: VERRECCHIA_EARLY_RESPONSE_TO_TGFB1
Profile of the Running ES Score & Positions of GeneSet Members on the Rank Ordered List

Gene Set enrichment of signatures associated with Neuronal function

Table: GSEA Results Summary

Dataset	GlutaVSGlutaCandy_Anova_GSEA_Anova_GSEA.Glutamate_vs_Glutamate+Candesartan.cls #Glutamate_versus_Glutamate+Candesartan.Glutamate_vs_Glutamate+Candesartan.cls #Glutamate_versus_Glutamate+Candesartan_repos
Phenotype	Glutamate_vs_Glutamate+Candesartan.cls#Glutamate_versus_Glutamate+Candesartan_repos
Upregulated in class	Glutamate+Candesartan
GeneSet	NEUROTRANSMITTER_RECEPTOR_ACTIVITY
Enrichment Score (ES)	-0.5032236
Normalized Enrichment Score (NES)	-1.8555192
Nominal p-value	0.0
FDR q-value	0.02209134
FWER p-Value	0.015

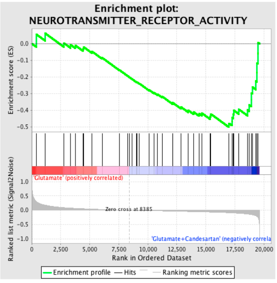

Fig 1: Enrichment plot: NEUROTRANSMITTER_RECEPTOR_ACTIVITY
Profile of the Running ES Score & Positions of GeneSet Members on the Rank Ordered List

Table: GSEA Results Summary

Dataset	GlutaVSGlutaCandy_Anova_GSEA_Anova_GSEA.Glutamate_vs_Glutamate+Candesartan.cls #Glutamate_versus_Glutamate+Candesartan.Glutamate_vs_Glutamate+Candesartan.cls #Glutamate_versus_Glutamate+Candesartan_repos
Phenotype	Glutamate_vs_Glutamate+Candesartan.cls#Glutamate_versus_Glutamate+Candesartan_repos
Upregulated in class	Glutamate+Candesartan
GeneSet	NEUROTRANSMITTER_BINDING
Enrichment Score (ES)	-0.46987364
Normalized Enrichment Score (NES)	-1.7600645
Nominal p-value	0.0
FDR q-value	0.027641125
FWER p-Value	0.036

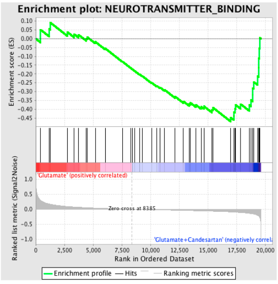

Fig 1: Enrichment plot: NEUROTRANSMITTER_BINDING
Profile of the Running ES Score & Positions of GeneSet Members on the Rank Ordered List

Table: GSEA Results Summary

Dataset	GlutaVSGlutaCandy_Anova_GSEA_Anova_GSEA.Glutamate_vs_Glutamate+Candesartan.cls #Glutamate_versus_Glutamate+Candesartan.Glutamate_vs_Glutamate+Candesartan.cls #Glutamate_versus_Glutamate+Candesartan_repos
Phenotype	Glutamate_vs_Glutamate+Candesartan.cls#Glutamate_versus_Glutamate+Candesartan_repos
Upregulated in class	Glutamate+Candesartan
GeneSet	SYNAPSE_ORGANIZATION_AND_BIOGENESIS
Enrichment Score (ES)	-0.7423028
Normalized Enrichment Score (NES)	-2.218165
Nominal p-value	0.0
FDR q-value	0.0
FWER p-Value	0.0

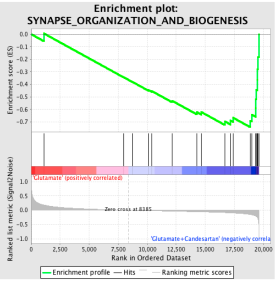

Fig 1: Enrichment plot: SYNAPSE_ORGANIZATION_AND_BIOGENESIS
Profile of the Running ES Score & Positions of GeneSet Members on the Rank Ordered List
